# Interaction models matter: an efficient, flexible computational framework for model-specific investigation of epistasis

**DOI:** 10.1186/s13040-024-00358-0

**Published:** 2024-02-28

**Authors:** Sandra Batista, Vered Senderovich Madar, Philip J. Freda, Priyanka Bhandary, Attri Ghosh, Nicholas Matsumoto, Apurva S. Chitre, Abraham A. Palmer, Jason H. Moore

**Affiliations:** 1 Department of Computational Biomedicine, Cedars-Sinai Medical Center, 700 N San Vicente Blvd., Pacific Design Center, Guite G540, West Hollywood, CA 90069 USA; 2 Chapel Hill, NC USA; 3 Department of Psychiatry, University of California, San Diego, 9500 Gilman Dr., Mailcode: 0667, La Jolla, CA 92093-0667 USA; 4 Institute for Genomic Medicine, University of California, San Diego, 9500 Gilman Dr., Mailcode: 0667, La Jolla, CA 92093-0667 USA

**Keywords:** Epistasis, Interaction model, Partial correlation, Linear regression, GWAS, XOR

## Abstract

**Purpose:**

Epistasis, the interaction between two or more genes, is integral to the study of genetics and is present throughout nature. Yet, it is seldom fully explored as most approaches primarily focus on single-locus effects, partly because analyzing all pairwise and higher-order interactions requires significant computational resources. Furthermore, existing methods for epistasis detection only consider a Cartesian (multiplicative) model for interaction terms. This is likely limiting as epistatic interactions can evolve to produce varied relationships between genetic loci, some complex and not linearly separable.

**Methods:**

We present new algorithms for the interaction coefficients for standard regression models for epistasis that permit many varied models for the interaction terms for loci and efficient memory usage. The algorithms are given for two-way and three-way epistasis and may be generalized to higher order epistasis. Statistical tests for the interaction coefficients are also provided. We also present an efficient matrix based algorithm for permutation testing for two-way epistasis. We offer a proof and experimental evidence that methods that look for epistasis only at loci that have main effects may not be justified. Given the computational efficiency of the algorithm, we applied the method to a rat data set and mouse data set, with at least 10,000 loci and 1,000 samples each, using the standard Cartesian model and the XOR model to explore body mass index.

**Results:**

This study reveals that although many of the loci found to exhibit significant statistical epistasis overlap between models in rats, the pairs are mostly distinct. Further, the XOR model found greater evidence for statistical epistasis in many more pairs of loci in both data sets with almost all significant epistasis in mice identified using XOR. In the rat data set, loci involved in epistasis under the XOR model are enriched for biologically relevant pathways.

**Conclusion:**

Our results in both species show that many biologically relevant epistatic relationships would have been undetected if only one interaction model was applied, providing evidence that varied interaction models should be implemented to explore epistatic interactions that occur in living systems.

**Supplementary Information:**

The online version contains supplementary material available at 10.1186/s13040-024-00358-0.

## Background

Epistasis is challenging to detect yet likely widespread and integral in biology. Evidence for epistasis has been discovered in a host of biological systems and phenotypes including mandible size in mice [1], cardiovascular disease susceptibility [2], coronary artery restenosis [3], cystic fibrosis [4, 5], and sporadic breast cancer [6] in humans, and most recently and robustly in two studies investigating non-additive genetic effects in yeast [7, 8]. The yeast studies have collectively identified thousands of epistatic two-way and three-way interactions that vary across lineages and growth conditions. Additionally, these studies identify large epistatic hubs that are involved in most interactions detected. In the most recent study in yeast, non-additive effects accounted for one-third of the broad-sense heritability [8]. These studies provide strong evidence that epistasis accounts for a large portion of the non-additive genetic variation observed across biology.

Since loci may contribute to phenotypes through non-linear interactions, epistasis may account for genetic variation not explained by single-locus approaches. Indeed, it has been shown that the main effect of one single nucleotide polymorphism (SNP) can significantly change when the allele frequencies in a second SNP are altered [9]. Examples of where epistasis may have a crucial role include biomolecular interactions in gene regulation, signal transduction, and biochemical networks [10–12]. Thus, many phenotypes can be viewed as the result of vast interconnected biological networks and systems [13–15]. These biological systems likely arise to form compensatory networks that aid in buffering against genetic and environmental change (i.e., canalization) [16–19]. It is likely that, at the core of these networks, interactions among polymorphisms from multiple pathways exist and are integral to organismal development, homeostasis, and survival [17].

Robust methodologies aimed at detecting and describing statistical epistasis are required to investigate genotype-phenotype associations and disease susceptibility [11]. Since experiments that could biologically validate detected statistical epistatic interactions are rare [13–15], the development of these methodologies will assist in initiating further scientific exploration. In this work, we examine how to efficiently compute linear regression models for epistasis that permit varied encodings of the interactions of loci and provide statistical evidence for epistasis. Recent computational and theoretical work has presented a new way to calculate each of the coefficients of a linear regression model [20]. In this work, we aim to demonstrate the usefulness and practical significance of the closed forms in general and more specifically in genetic studies and in particular, epistasis. We present algorithms for providing statistical evidence for two-way and three-way epistasis using standard models for epistasis using these closed forms. These algorithms may be used efficiently on subsets of loci or genome-wide, are entirely parallelizable, provide statistical tests, and permit flexibility in encoding the interaction models of loci. Many methods for interaction terms only consider the Cartesian model that multiplies the genotype vectors at two loci. In our method, any function may be applied to interacting genotypes and we demonstrate this using both the Cartesian interaction model and the exclusive-or (XOR) interaction model. We also discuss how these algorithms may be generalized for higher order interactions. Permitting many types of encodings for interaction models allows for complexity to be included in the traditional statistical models for epistasis and for biologists to use a variety of interaction models to investigate epistasis.

As specific examples, we apply our algorithms to detect statistical evidence for two-way and three-way interactions using the Cartesian and XOR interaction models on the phenotype of body mass index (BMI) in real data sets from rats (*Rattus norvegicus*) from a genome-wide association study (GWAS) [21–23] investigating obesity-related traits and from mice (*Mus musculus*) from Wellcome Trust [24, 25]. For both data sets we use approximately 10,000 SNPs for two-way epistasis detection using both interaction models.

### Computational challenges in detecting epistasis

Methods to explore genotype-phenotype associations and detect epistatic interactions are often computationally intensive and may completely ignore non-additive effects. Single-locus analyses like GWAS can detect strong main effects, but face difficulties when applied to combinations of variables for many reasons [14, 15]. The first is that multi-locus genotype (MLG) combinations have smaller representative samples compared to the original data set due to low minor allele frequencies in some loci. Second, in most approaches that attempt to model interactions using linear models, interactions are only considered when significant main effects of variables are identified [14, 15]. Although it is tempting to expect loci with significant main effects to also be involved in interactions, there is no statistical justification for this. Third, while linear models are efficient in detecting and estimating the main effects of variables, they are typically less effective at identifying interaction effects, which often require more complex modeling approaches [26–28]. Fourth, many linear model approaches construct interaction terms using the Cartesian product for ease of computation whereas other models of interaction may also be plausible. We expand more on this in a following section. Finally, when considering higher order interactions, as the number of loci in k-wise combinations increases, the number of variables in the standard regression models increases exponentially and the total number of sets of loci of size *k* to consider of all *n* possible loci increases polynomially as more loci are considered. This exhaustive search space creates issues with computational tractability as investigating pairwise and higher-order interactions becomes extremely difficult to achieve efficiently. Given these challenges, some of which are inherent in exhaustively considering all possible subsets for k-wise interactions in a set of n loci, many methods apply various techniques to reduce this search space or to exploit parallelism in underlying matrix libraries for computational efficiency.

There are many techniques to reduce the search space for epistasis algorithms. One example, the multifactor dimensionality reduction (MDR) technique finds MLGs that have high or low association with disease and defines new variables that explain the relationship of both loci [6, 29, 30]. MDR can be combined with other machine learning methods and has been extended to handle population structure [31]. In an approach that bypasses the conventional search space of epistasis algorithms, Crawford et al. developed the “MArginal ePIstasis Test” (MAPIT) method that measures the marginal epistatic effect of a single loci against all other loci all at once [32]. MAPIT can be used as a way to screen for all loci that have significant marginal epistastic effects for subsequent tests to see which pairs of loci may be involved in epistasis. More comprehensive recent surveys on epistasis are offered by Ogbunugafor and Scarpino on higher order epistasis [33], Niel et al. on statistical and computational challenges of varied approaches [34], and Russ et al. for performance comparison of many varied epistasis detection methods [35].

In exhaustive methods for statistical epistasis for discrete (case/control) phenotypes, a common approach to make the problem more computationally tractable is to use contingency tables for approximating pairwise epistasis. One of the most commonly used software packages for applying the standard pairwise epistasis regression models is PLINK [36]. In PLINK, pairs of loci are scanned using an approximate method that evaluates the Z-scores for the odds ratio at the loci between cases and controls or BOOST that uses bitwise operations for calculating the likelihood ratio test [37]. It is then possible to apply the entire regression to the pairs of loci that pass the screening stage. Zhang et al. use minimum spanning trees to update contingency tables for test statistics for evaluating pairs of loci for epistasis in the TEAM algorithm [38]. These aforementioned approaches work for pairwise epistasis. Bayat et al. offer the BitEpi method for handling up to four-way epistasis using bit efficient counting for contingency tables, an entropy metric to calculate the interaction effect, and permutations for *p*-value calculations [39].

Another approach to ease the computational burden for exhaustive methods for statistical epistasis is to exploit the parallelism from hardware architectures or underlying matrix operations. Schupbach et al. present FastEpistasis as an extension to PLINK that for quantitative phenotypes calculates the interaction term for a pair of loci by using the QR decomposition to solve the ordinary least squares [40]. The efficiency from their method is from the parallelism of the calculations and the hardware architectures. Zhu and Fang offer the method MatrixEpistasis that evaluates the standard regression model for epistasis for the interaction term by computing the residuals of the linear regression model between the phenotype and loci and the residuals for the regression model between the interaction and loci [41]. Like our method, some of the efficiency of MatrixEpistasis is achieved by only focusing on the interaction term and corresponding test statistic, but the effectiveness of their method also relies on the efficiency of underlying matrix operations and accordingly the method can only handle Cartesian model encoding of the interaction term. The work of Zhu and Fang also considered covariate adjustment, but that may be done before applying our algorithm as is essentially done in their implementation and commonly done in practice as well. Unlike these other methods, our method permits many different encodings for the interaction model, thus potentially introducing some non-linearity. Our method also explicitly deals with higher order epistasis. The method we present is the result of new computational and algorithmic insight for solving the ordinary least squares that expresses the estimates in closed forms. As a result, our method is also very efficient in terms of memory and entirely parallelizable for testing each subset of loci for epistasis.

## Methods

### Models for epistasis

At the center of modeling epistasis is regression and we may now consider whether locus *i* and locus *j* have an epistatic interaction, $I_{i,j}$, affecting the phenotype, *p*, by considering their interaction effect term, $\beta _3$ :

$$\begin{aligned} p = \beta _0 + \beta _1 g_i + \beta _2 g_j+ \beta _3 I_{i,j} \end{aligned}$$

We may remove the intercept and simplify the computation of the model by subtracting off the means of the variables, i.e. $\tilde{g_i} = g_i-\bar{g}_i$, $\tilde{p} = p - \bar{p}$ and $\widetilde{I_{i,j}} = I_{i,j} - \bar{I}_{i,j}$:

$$\begin{aligned} \tilde{p} = \beta _1 \tilde{g_i} + \beta _2 \tilde{g_j}+ \beta _3 \widetilde{I_{i,j}} \end{aligned}$$

We are only concerned with $\beta _3$, the coefficient for the interaction term, $\widetilde{I_{i,j}}$, in the model because it represents the partial correlation between the phenotype and the interaction term given both loci. It is important to note that there is flexibility in this model in how the interaction is encoded. Although it is common to multiply values of the loci for each sample as is commonly done for the Cartesian model for ease of computation, many different encoding models may be used as we demonstrate for the XOR model (snp1 *mod *2 + snp2 *mod* 2)*mod* 2) (S2 File). While the Fisher t-statistics for hypothesis testing can be applied (e.g. [41]), a direct *T* test for a regression coefficient may also be applied instead.

The model for epistasis may be generalized to higher order interactions. For $k-$order interactions between a set of *k* loci, $g_i$ for $1 \le i \le k$, we must consider the interactions of all subsets of the loci from the empty set to the entire set of all *k* in a manner reminiscent of applying the binomial theorem where the coefficient is the interaction rather than binomial coefficient. For the epistatic interaction of a subset, we will use the variable $I_s$ where *s* is the subset. An appropriate function, *f*, for the interaction model encoding, such as XOR or Cartesian, may be applied to the sets as well as the interaction term of the entire set of *k* loci. All variables may then also be centered by subtracting off the means. The model for the epistatic interactions affecting phenotype *p* may be expressed as follows:

$$\begin{aligned} \widetilde{p} = \left[ \sum _{i=1}^{k-1}\sum _{s \subset \{g_1, \ldots , g_k\}, |s|=i}\beta _s f(\widetilde{ X_s}) \right] + \beta _{\{g_1, \ldots , g_k\}} \widetilde{I_{\{g_1, \ldots , g_k\}}} \end{aligned}$$

The model that we will consider for 3-way epistasis for loci $g_1, g_2, g_3$ is accordingly after centering the variables:

$$\begin{aligned} \tilde{p} = \beta _1\tilde{g_1} + \beta _2\tilde{g_2}+ \beta _3\tilde{g_3}+ \beta _{\{1,2\}}\tilde{I}_{\{1,2\}} +\beta _{\{1,3\}}\tilde{I}_{\{1,3\}} + \beta _{\{2,3\}}\tilde{I}_{\{2,3\}} + \beta _{\{1,2,3\}}\tilde{I}_{\{1,2,3\}} \end{aligned}$$

For detecting epistasis, the interaction term of interest is $\beta _{\{1,2,3\}}$ for 3-way epistasis and $\beta _{\{g_1, \ldots , g_k\}}$ for k-way epistasis. It is important to note that for $k-$way epistasis the regression must include $O(2^k)$ variables for each set of *k* loci and that to check for all possible $k-$wise interactions between *n* loci, there are $O(n^k)$ sets that must be checked for the given model. For a given set of *k* loci, we explore how it is not necessary to find the interaction coefficients for all $2^k-1$ variables and may focus only on the final coefficient of interaction along with more efficient and powerful statistical tests of interaction. We give algorithms explicitly for $k=2,3$ and consider how these may be generalized.

### Estimation and statistical tests for interaction

The conditional association between the phenotype, *p*, and the interaction term, *I*, while conditioning upon the main effects of the loci, $${\textbf {Z}} = \{g_i,g_j\}$$, is the epistasis interaction. This may be computed in multiple ways. The first way is to compute the partial correlation coefficient between *p* and *I*, as the dot product between two standardized residuals: the residuals for the regression model between the phenotype and the loci and the residuals for the regression model between the interaction and loci. This approach requires the computation of two vectors of residuals estimated by two linear regression models. The first linear regression model is between the phenotype and loci, $$p \sim \beta _0 + {\textbf {Z}}$$ and the second model is between the interaction and loci, $$I \sim \beta _0 + {\textbf {Z}}$$, as was done in the work of Zhu and Zhang [41]. The standardized residual of the linear regression model between the phenotype and loci, $$p \sim \beta _0 + {\textbf {Z}}$$, is $$resid_p = p - \hat{\beta }_0^* - \hat{\beta }_1^* g_i - \hat{\beta }_2^* g_j$$ where the $$\hat{\beta }^*$$ are the estimates from the model. The standardized residual of the regression between the interaction and loci, $$I \sim \beta _0 + {\textbf {Z}}$$, is $$resid_I = I - \hat{\beta }_0^{**} - \hat{\beta }_1^{**} g_i - \hat{\beta }_2^{**} g_j$$ where again the $$\hat{\beta }^{**}$$ are the estimates from the model. This means two separate regression models are applied as was done in the work of Zhu and Zhang [41]. The partial correlation coefficient between *p* and *I* is thus *r* :

$$\begin{aligned} r = \left\langle \frac{resid_p}{\sqrt{<resid_p,resid_p>}},\frac{resid_I}{\sqrt{<resid_I,resid_I>}}\right\rangle \end{aligned}$$

The second way is to compute a single regression coefficient, the corresponding regression coefficient, $\hat{\beta }_3$ in the model between the phenotype, loci, and interaction term: $p \sim \beta _0 + \beta _2 g_1 + \beta _2 g_2 + \beta _3 I$. Both measures are variants of each other, as a computed $r = 0$ will necessarily imply a computed $\hat{\beta }_3 = 0$ and vice versa. This is due to Yule’s equivalence formula [42],

$$\begin{aligned} \hat{\beta }_3 = r \cdot \frac{\sqrt{<resid_p,resid_p>}}{\sqrt{<resid_I,resid_I>}} \quad \text {or the equivalent form}\quad \hat{\beta }_3 = \frac{<resid_p,resid_I>}{<resid_I,resid_I>} \end{aligned}$$

We know now that $<resid_p,resid_I> = <\textbf{p},resid_I>$ and know a direct way to estimate $\hat{\beta }_3$ and each of the other coefficients that is the crux of our algorithm [20]. Consider the estimation of the model assuming all variables are mean centered:

$$\begin{aligned} \widetilde{\textbf{p}} = \beta _1 \widetilde{\textbf{g}_i} + \beta _2 \widetilde{\textbf{g}_j} +\beta _3 \widetilde{\textbf{I}} \end{aligned}$$

We have by considering the residuals of each loci with the interaction term an expression for the interaction effect term, $\beta _3$:

$$\begin{aligned} \hat{\beta }_3 = \frac{ \left\langle \widetilde{\textbf{p}} , \widetilde{\textbf{I}} - \frac{ \left\langle \widetilde{\textbf{g}_i} , \widetilde{\textbf{I}}\right\rangle }{ \left\langle \widetilde{\textbf{g}_i} , \widetilde{\textbf{g}_i} \right\rangle } \widetilde{\textbf{g}_i} \right\rangle - \frac{ \left\langle \widetilde{\textbf{I}} , \widetilde{\textbf{g}_j} - \frac{ \left\langle \widetilde{\textbf{g}_i} , \widetilde{\textbf{g}_j}\right\rangle }{ \left\langle \widetilde{\textbf{g}_i} , \widetilde{\textbf{g}_i} \right\rangle } \widetilde{\textbf{g}_i} \right\rangle }{ \left\langle \widetilde{\textbf{g}_j} , \widetilde{\textbf{g}_j} \right\rangle - \frac{ \left\langle \widetilde{\textbf{g}_i} , \widetilde{\textbf{g}_j} \right\rangle ^2}{ \left\langle \widetilde{\textbf{g}_i} , \widetilde{\textbf{g}_i} \right\rangle } } \left\langle \widetilde{\textbf{p}} , \widetilde{\textbf{g}_j} - \frac{ \left\langle \widetilde{\textbf{g}_i} , \widetilde{\textbf{g}_j} \right\rangle }{ \left\langle \widetilde{\textbf{g}_i} , \widetilde{\textbf{g}_i} \right\rangle } \widetilde{\textbf{g}_i} \right\rangle }{ \left\langle \widetilde{\textbf{I}} , \widetilde{\textbf{I}} \right\rangle - \frac{ \left\langle \widetilde{\textbf{g}_i} , \widetilde{\textbf{I}} \right\rangle ^2 }{ \left\langle \widetilde{\textbf{g}_i} , \widetilde{\textbf{g}_i} \right\rangle } - \frac{ \left( \left\langle \widetilde{\textbf{g}_j} , \widetilde{\textbf{I}} \right\rangle - \frac{ \left\langle \widetilde{\textbf{g}_i} , \widetilde{\textbf{g}_j}\right\rangle }{ \left\langle \widetilde{\textbf{g}_i} , \widetilde{\textbf{g}_i} \right\rangle } \left\langle \widetilde{\textbf{g}_i} , \widetilde{\textbf{I}}\right\rangle \right) ^2 }{ \left\langle \widetilde{\textbf{g}_j} , \widetilde{\textbf{g}_j} \right\rangle - \frac{ \left\langle \widetilde{\textbf{g}_i} , \widetilde{\textbf{g}_j} \right\rangle ^2}{ \left\langle \widetilde{\textbf{g}_i} , \widetilde{\textbf{g}_i} \right\rangle } } } \end{aligned}$$

In particular, we may write the vector of partial residuals of the interaction term with each loci, $resid_{I}$, directly as

$$\begin{aligned} resid_I = \widetilde{\textbf{I}} - \frac{ \left\langle \widetilde{\textbf{I}} , \widetilde{\textbf{g}_i}\right\rangle }{ \left\langle \widetilde{\textbf{g}_i} ,\widetilde{\textbf{g}_i} \right\rangle } \widetilde{\textbf{g}_i} - \frac{ \left\langle \widetilde{\textbf{I}} , \widetilde{\textbf{g}_j} - \frac{ \left\langle \widetilde{\textbf{g}_i} ,\widetilde{\textbf{g}_j}\right\rangle }{ \left\langle \widetilde{\textbf{g}_i} , \widetilde{\textbf{g}_i} \right\rangle } \widetilde{\textbf{g}_i} \right\rangle }{ \left\langle \widetilde{\textbf{g}_j} , \widetilde{\textbf{g}_j} \right\rangle - \frac{ \left\langle \widetilde{\textbf{g}_i} , \widetilde{\textbf{g}_j} \right\rangle ^2}{ \left\langle \widetilde{\textbf{g}_i} , \widetilde{\textbf{g}_i} \right\rangle } } \left( \widetilde{\textbf{g}_j} - \frac{ \left\langle \widetilde{\textbf{g}_i} , \widetilde{\textbf{g}_j} \right\rangle }{ \left\langle \widetilde{\textbf{g}_i} , \widetilde{\textbf{g}_i} \right\rangle } \widetilde{ \textbf{g}_i} \right) \end{aligned}$$

while in a similar way we may write the vector of partial residuals of the phenotype with each loci, $resid_{p}$, directly as

$$\begin{aligned} resid_p = \widetilde{\textbf{p}} - \frac{ \left\langle \widetilde{\textbf{p}} , \widetilde{\textbf{g}_i}\right\rangle }{ \left\langle \widetilde{\textbf{g}_i} ,\widetilde{\textbf{g}_i} \right\rangle } \widetilde{\textbf{g}_i} - \frac{ \left\langle \widetilde{\textbf{p}} , \widetilde{\textbf{g}_j} - \frac{ \left\langle \widetilde{\textbf{g}_i} ,\widetilde{\textbf{g}_j}\right\rangle }{ \left\langle \widetilde{\textbf{g}_i} , \widetilde{\textbf{g}_i} \right\rangle } \widetilde{\textbf{g}_i} \right\rangle }{ \left\langle \widetilde{\textbf{g}_j} , \widetilde{\textbf{g}_j} \right\rangle - \frac{ \left\langle \widetilde{\textbf{g}_i} , \widetilde{\textbf{g}_j} \right\rangle ^2}{ \left\langle \widetilde{\textbf{g}_i} , \widetilde{\textbf{g}_i} \right\rangle } } \left( \widetilde{\textbf{g}_j} - \frac{ \left\langle \widetilde{\textbf{g}_i} , \widetilde{\textbf{g}_j} \right\rangle }{ \left\langle \widetilde{\textbf{g}_i} , \widetilde{\textbf{g}_i} \right\rangle } \widetilde{ \textbf{g}_i} \right) \end{aligned}$$

As a result we may rewrite the interaction effect term, $\hat{\beta }_3$. It may be expressed in terms of the ratio of two dot products between the dependent variable and the partial residuals and between the independent variable *I* and partial residuals as follows:

$$\begin{aligned} \hat{\beta }_3 = \frac{\left\langle \widetilde{\textbf{p}}, resid_I\right\rangle }{\left\langle \widetilde{\textbf{I}}, resid_I\right\rangle } \end{aligned}$$

As for hypotheses test, we test the null hypotheses

$$\begin{aligned} H_0: \text {The conditional association between}\ I\ \text {and}\ p \, = 0 \end{aligned}$$

against the alternative

$$\begin{aligned} H_1: \text {The conditional association between}\ I\ \text {and}\ p \, \ne 0 \end{aligned}$$

This calls for multiple testing correction to protect against the inflated Type I error and we use the Benjamini Hochberg false discovery rate (FDR) controlling procedure for this. The statistical test will be a T-test with $n-v-1$ as degrees of freedom where *n* is the number of samples and *v* is the number of parameters in the model.

Zhu and Zhang address the partial correlation between the interaction term *I* and the phenotype *p*, and compute it using the dot product of the residuals [41]. As for a statistical test to test for null value for the association, the Fisher approximate test is then used [43, pp. 26 ]:

$$\begin{aligned} T_{Fisher} = \sqrt{n-v-1}\frac{r}{\sqrt{1-r^2}} \end{aligned}$$

The T test statistic for testing the null hypothesis against the alternative will be in terms of successive residuals: the partial residual, $resid_p$ aforementioned, and the global residual, $resid= p - \hat{\beta }_0 - \hat{\beta }_1 g_i - \hat{\beta }_2 g_j -\hat{\beta }_3 I$, for $p \sim \beta _0 + \beta _2 g_1 + \beta _2 g_2 + \beta _3 I$ after adding *I* to the model. We observe that we may calculate the mean sum of squares or the mean square error, MSE, using the global residual, that is,

$$\begin{aligned} MSE = \frac{<resid,resid>}{(n-v-1)} = \frac{<\widetilde{\textbf{p}},resid>}{(n-v-1)} \end{aligned}$$

As a result we may calculate the test statistic as well:

$$\begin{aligned} T(\beta _3) = \hat{\beta }_3 \cdot \sqrt{\frac{< \widetilde{\textbf{I}},resid_I>}{MSE}} \end{aligned}$$

$$\begin{aligned} T(\beta _3) = \sqrt{n-v-1}\frac{<\widetilde{\textbf{p}},resid_I>}{\sqrt{<\widetilde{\textbf{I}}, resid_I> \cdot < \widetilde{\textbf{p}},resid>}} \end{aligned}$$

Both of the T-tests above require the computation of the $<\widetilde{\textbf{p}},resid_p> (= <resid_p,resid_p>)$ or the MSE (for modeling *p*) or where it is possible to verify that

$$\begin{aligned} MSE \cdot (n-v-1) =<\widetilde{\textbf{p}},resid> =<\widetilde{\textbf{p}},resid_p> - \hat{\beta }_3 \cdot <\widetilde{\textbf{p}},\widetilde{\textbf{I}}> . \end{aligned}$$

### Algorithms for epistasis detection

We may now give an algorithm for computing the interaction coefficient and test statistic for epistasis for two loci, $g_i$ and $g_j$. We may encode their interaction, $I_{i,j}$ using any type of epistasis that may be suspected and often by default the Cartesian encoding (or product) is used. We will use the centered variables for our algorithm where the means have been subtracted, i.e. $\tilde{g_i} = g_i-\bar{g}_i$, $\tilde{p} = p - \bar{p}$ and $\tilde{I_{i,j}} = I_{i,j} - \bar{I}_{i,j}$. The algorithm residualizes the first locus from the second and then each loci from their interaction in order. The algorithm does the same in residualizing each loci from the phenotype in order. Finally to calculate the residuals of the entire model, we residualize the interaction from the phenotype. In the case of pairwise epistasis, the algorithm only requires the centered variables to calculate several vector dot products and vector additions (subtractions). In the exposition given below, we calculate the direct t-test statistic for the regression coefficient. Let the number of samples for each loci and the phenotype be *m*.

**Algorithm 1:**
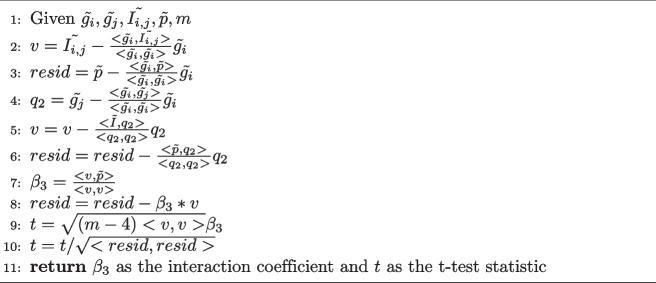
Interaction Coefficient for Pairwise Epistasis

The algorithm is linear in the number of samples *m*. However, if the aim is to test all possible pairwise epistasis for *n* loci, this will still be an $O(n^2m)$ algorithm. The flexibility here is to test any pair of loci for epistasis with a variety of test statistics and a variety of possible interaction model encodings. There is also the practical concern regarding floating point representation in implementations if any of the vector norms or norms squared, such as the norm of the residuals (in line 10), are close to zero. In some implementations this could raise an error because of division by zero, for example, because of limitations in floating point representations. In this case we recommended that any such errors that arise, while not errors in the algorithm or implementation themselves, but merely limitations of floating point representations, be logged for further inspection as we have done in our experiments.

We will now consider the algorithm for 3-way epistasis for a set of loci. While technically the algorithm is still linear in the number of samples since $k=3$ is a constant, we begin to see more of the effects on the complexity of the number of variables in the model. For the case of 3-way epistasis, there are only 7 variables, but more generally there are $O(2^k)$ variables in models for $k-$way epistasis. Also, the algorithm is quadratic in the number of variables while linear in the number of samples. Thus, the more general case has complexity $O(2^{2k}m)$ for a single set of *k* loci. The models themselves introduce a number of variables exponential in *k* and for all possible sets of *k* loci from *n* the complexity is $O(n^k2^{2k}m)$, so there is a need to prune the space of loci to check and variables in the models.

However, first we present the algorithm for 3-way epistasis detection, and for the sake of exposition, mention how it can be generalized to $k-$wise epistasis with the caveats we have given for its time complexity. For the encoding of all interaction models, we assume that a Cartesian encoding (or product of the loci) are used. We assume that we have three loci, $g_1,g_2, g_3$ and the phenotype, *p* as well as their encodings and they are all centered (with their means subtracted from them). First, we construct an 8 by *m* matrix *Q* that will be used to residualize each variable against each other in the order given. For ease of exposition, we will index the columns of *Q* for each variable starting at 1 where 1 will be the index for $\tilde{g_1}$, 7 for $\tilde{I_{\{1,2,3\}}}$and 8 for $\tilde{p}$. It is important to note that we are overwriting Q. This is important to mention since it clarifies not only the correctness of the algorithm, but highlights the space efficiency of these algorithms. Once the intermediate matrix for the 8 (or more generally $2^k$) variables for the model is constructed, for which only read only access of the entire data matrix is required, no additional access to the data matrix is needed. Additionally, only additional constant space for several dot products and to hold the interaction coefficient and test statistic are needed.

**Algorithm 2:**
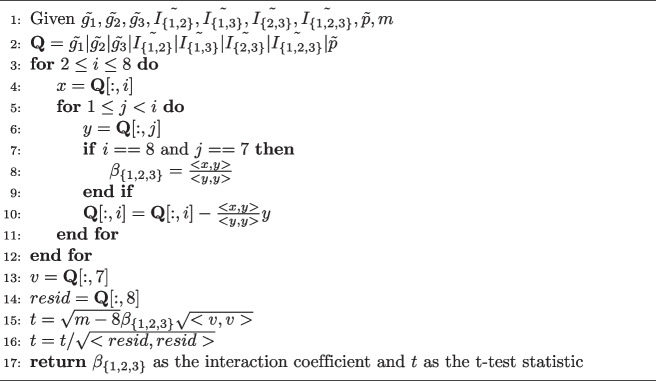
Interaction Coefficient for 3-way Epistasis

To generalize the 3-way epistasis algorithm to be a $k-$wise epistasis algorithm, we need to first enumerate the $2^k$ variables of the model and center them by subtracting off their means to construct $\textbf{Q}$. Then it is necessary to residualize the variables by changing 8 to the value $2^k$ on line 3. To get the interaction coefficient for all *k* loci, $\beta _{\{1,\ldots ,k\}}$, on line 7 checks that $i == 2^k$ and $j = 2^k-1$. To calculate the t-test statistic, on line 13 set $v=\textbf{Q}[:,2^k-1]$ and on line 14 $resid= \textbf{Q}[:,2^k]$ and adjust the degrees of freedom on line 15. If we have a set of loci of interest, this algorithm and its generalization can calculate their interaction coefficient and test statistic efficiently. However, as we noted if we do not have a known set of loci a priori, then there is work to be done to prune what would be the exhaustive search space as we will consider in the next section.

The extension of the algorithms for permutation tests can be easily done by permutations of the dependent variable $\textbf{p}$ while computing $resid_I$, $resid_p$ and in the case of a pairwise epistasis, $\hat{\beta }_3$ as

$$\begin{aligned} \hat{\beta }_3 = \frac{\left\langle \widetilde{\textbf{p}}, resid_I\right\rangle }{\left\langle \widetilde{\textbf{I}}, resid_I\right\rangle } \end{aligned}$$

$$\begin{aligned} resid_I = \widetilde{\textbf{I}} - \frac{ \left\langle \widetilde{\textbf{I}} , \widetilde{\textbf{g}_i}\right\rangle }{ \left\langle {\textbf{g}_i} ,\widetilde{\textbf{g}_i} \right\rangle } \widetilde{\textbf{g}_i} - \frac{ \left\langle \widetilde{\textbf{I}} , \widetilde{\textbf{g}_j} - \frac{ \left\langle \widetilde{\textbf{g}_i} ,\widetilde{\textbf{g}_j}\right\rangle }{ \left\langle \widetilde{\textbf{g}_i} , \widetilde{\textbf{g}_i} \right\rangle } \widetilde{\textbf{g}_i} \right\rangle }{ \left\langle \widetilde{\textbf{g}_j} , \widetilde{\textbf{g}_j} \right\rangle - \frac{ \left\langle \widetilde{\textbf{g}_i} , \widetilde{\textbf{g}_j} \right\rangle ^2}{ \left\langle \widetilde{\textbf{g}_i} , \widetilde{\textbf{g}_i} \right\rangle } } \left( \widetilde{\textbf{g}_j} - \frac{ \left\langle \widetilde{\textbf{g}_i} , \widetilde{\textbf{g}_j} \right\rangle }{ \left\langle \widetilde{\textbf{g}_i} , \widetilde{\textbf{g}_i} \right\rangle } \widetilde{ \textbf{g}_i} \right) \end{aligned}$$

and

$$\begin{aligned} resid_p = \widetilde{\textbf{p}} - \frac{ \left\langle \widetilde{\textbf{p}} , \widetilde{\textbf{g}_i}\right\rangle }{ \left\langle {\textbf{g}_i} ,\widetilde{\textbf{g}_i} \right\rangle } \widetilde{\textbf{g}_i} - \frac{ \left\langle \widetilde{\textbf{p}} , \widetilde{\textbf{g}_j} - \frac{ \left\langle \widetilde{\textbf{g}_i} ,\widetilde{\textbf{g}_j}\right\rangle }{ \left\langle \widetilde{\textbf{g}_i} , \widetilde{\textbf{g}_i} \right\rangle } \widetilde{\textbf{g}_i} \right\rangle }{ \left\langle \widetilde{\textbf{g}_j} , \widetilde{\textbf{g}_j} \right\rangle - \frac{ \left\langle \widetilde{\textbf{g}_i} , \widetilde{\textbf{g}_j} \right\rangle ^2}{ \left\langle \widetilde{\textbf{g}_i} , \widetilde{\textbf{g}_i} \right\rangle } } \left( \widetilde{\textbf{g}_j} - \frac{ \left\langle \widetilde{\textbf{g}_i} , \widetilde{\textbf{g}_j} \right\rangle }{ \left\langle \widetilde{\textbf{g}_i} , \widetilde{\textbf{g}_i} \right\rangle } \widetilde{ \textbf{g}_i} \right) \end{aligned}$$

They may be used in order to obtain the test statistic

$$\begin{aligned} T(\beta _3) = \sqrt{n-4}\frac{<\widetilde{\textbf{p}},resid_I>}{\sqrt{<\widetilde{\textbf{I}}, resid_I> \cdot < \widetilde{\textbf{p}},resid>}} \end{aligned}$$

In the case of pairwise epistasis

$$\begin{aligned} \left\langle \widetilde{\textbf{p}},resid \right\rangle = \left\langle \widetilde{\textbf{p}},resid_p\right\rangle - \hat{\beta }_3 \cdot \left\langle \widetilde{\textbf{p}},\widetilde{\textbf{I}}\right\rangle = \left\langle \widetilde{\textbf{p}},resid_p\right\rangle - \frac{\left\langle \widetilde{\textbf{p}}, resid_I\right\rangle }{\left\langle \widetilde{\textbf{I}}, resid_I\right\rangle } \cdot \left\langle \widetilde{\textbf{p}},\widetilde{\textbf{I}}\right\rangle . \end{aligned}$$

Note that $resid_p$, the dot product of the permuted phenotype, and the interaction term needs to be updated for each permutation. The interaction term and its residual may be reused for all permutations. The extension of the pairwise epistasis algorithm with permutation testing follows in Algorithm 3. The interaction coefficient, $\beta _3$, and T statistic are computed as in Algorithm 1. Algorithm 3 calculates the interaction coefficient and test statistic for each permuted phenotype. The *p*-value is the percentage of permuted phenotypes with test statistics at least as large as the test statistic for the original phenotype. We present the algorithm using matrix calculations on the permuted phenotype matrix, *P*, an *m* by *K* matrix, where *m* is the number of samples and *K* is the number of permutations. Each column of *P* is a permutation of the original phenotype vector, $\widetilde{\textbf{p}}$. There are several advantages to this approach in that calculating *P* once for all permutation tests permits efficient parallelization of the permutation testing. The permuted phenotype matrix may be divided into submatrices of columns of permutations if need be, especially for memory limitations. In this case, the calculation of the *p*-value would be modified to be done across the divisions (i.e., return the number of tests at least as large as original t-test in absolute value and calculate *p*-value after all computations are done). Second, using matrix operations permits implementations of the algorithm to exploit underlying parallelization and efficiency of matrix libraries such as BLAS and LAPACK used by NumPy in Python.

**Algorithm 3:**
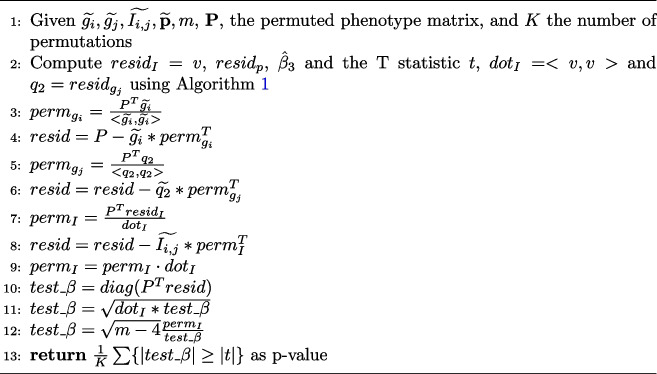
Permutation Test *P*-value for Pairwise Epistasis

It should be noted that permutation tests offer a robust alternative statistical test which is preferred in case there is suspicion that the required linear regression assumptions of nonlinearity or nonnormality fail to hold. Permutation tests are also known to control better the family wise error rate in the scope of the specific model under testing. As each permutation test is performed under a specific epistasis model, when considered altogether, for all pairs, the control of the global inflation of the Type I error is not assured. To determine if permutation tests notably affect our results compared to FDR correction alone, we implemented Algorithm 3 and applied it to perform 1000 permutations for pairwise epistasis detection using the XOR interaction model on the rat data set.

### Screening for higher order epistasis

We consider whether there is any mathematical justification from the models for epistasis to prune the search space of higher order interactions from lower order interactions or vice versa. For example, a common heuristic is to search the main effects for the pairwise epistasis and pairwise epistasis for 3-way epistasis. A variant of an approach that uses stages like this is used, for example, by Laurie et. al. [44].

Without loss of generality we are considering $k+1$ loci, $g_1, \ldots g_{k+1}$ and their interactions are all encoded using the Cartesian (product) model. If such justification exists, we may be able to show claims such that if there is a significant $k+1$-way interaction among $k+1$ loci, then all *k* interactions in the set of $k+1$ loci are significant. The contrapositive of this claim is more practically useful: If any *k*-wise interaction is not significant from the set of loci, then $k+1$-wise interaction is also not significant. The converse (i.e., if for a set of $k+1$ loci, all $k-$wise epistasis exist, then the loci have $k+1-$wise epistasis) is less plausible still. There is empirical evidence in the rat data set in this study (and many others) against both claims in considering only main effects and pairwise epistasis. Namely, there are main effect loci that are not in pairwise epistasis with each other; there are main effect loci in pairwise epistasis with a loci that is not a main effect, and there exists pairwise epistasis between loci that are not main effects.

Nevertheless we examine why these claims do not hold for the models used in epistasis. To do so, we consider two loci and their staged models for main effects and pairwise epistasis. Here we express the models simultaneously assuming the variables are centered. First we consider main effects:

$$\begin{aligned} \widetilde{p} = \beta _{1i}\widetilde{g_i} \qquad \text {or} \qquad \widetilde{p} =\beta _{1j}\widetilde{g_j} \end{aligned}$$

and pairwise epistasis

$$\begin{aligned} \widetilde{p} = \beta _{2i}\widetilde{g_i} + \beta _{2j}\widetilde{g_j}+ \beta _{ij}\widetilde{I_{i,j}} \end{aligned}$$

In terms of the coefficients in the models, the first claim is that at least one loci not being a main effect implies no pairwise epistasis: if $\hat{\beta }_{1i} = 0$ or $\hat{\beta }_{1j} = 0$, then $\hat{\beta }_{ij} = 0$. The concern here is really the inference needed regarding the coefficients of the pairwise model given knowledge of the coefficients in the main effects model. While having evidence $\hat{\beta }_{ij} = 0$ does not give us implications about $\hat{\beta }_{2j}$ or $\beta _{2i}$, knowing that either is 0 would give an implication regarding $\hat{\beta }_{ij} = 0$. In general, without loss of generality, recent closed forms for the ordinary least squares coefficients [20] give extensions to the formula of Yule [42],

$$\begin{aligned} \hat{\beta }_{1i} = \frac{< \widetilde{\textbf{p}}, \widetilde{\textbf{g}_i}>}{< \widetilde{\textbf{g}_i}, \widetilde{\textbf{g}_i}>} \qquad \text {and}\qquad \hat{\beta }_{1j} = \frac{< \widetilde{\textbf{p}}, \widetilde{\textbf{g}_j}>}{< \widetilde{\textbf{g}_j}, \widetilde{\textbf{g}_j}>} \end{aligned}$$

and for the pairwise epistasis model

$$\begin{aligned} \hat{\beta }_{ij} = \frac{ \left\langle \widetilde{\textbf{p}} , \widetilde{\textbf{I}_{ij}} - \frac{ \left\langle \widetilde{\textbf{g}_i} , \widetilde{\textbf{I}_{ij}}\right\rangle }{ \left\langle \widetilde{\textbf{g}_i} , \widetilde{\textbf{g}_i} \right\rangle } \widetilde{\textbf{g}_i} \right\rangle - \frac{ \left\langle \widetilde{\textbf{I}_{ij}} , \widetilde{\textbf{g}_j} - \frac{ \left\langle \widetilde{\textbf{g}_i} , \widetilde{\textbf{g}_j}\right\rangle }{ \left\langle \widetilde{\textbf{g}_i} , \widetilde{\textbf{g}_i} \right\rangle } \widetilde{\textbf{g}_i} \right\rangle }{ \left\langle \widetilde{\textbf{g}_j} , \widetilde{\textbf{g}_j} \right\rangle - \frac{ \left\langle \widetilde{\textbf{g}_i} , \widetilde{\textbf{g}_j} \right\rangle ^2}{ \left\langle \widetilde{\textbf{g}_i} , \widetilde{\textbf{g}_i} \right\rangle } } \left\langle \widetilde{\textbf{p}} , \widetilde{\textbf{g}_j} - \frac{ \left\langle \widetilde{\textbf{g}_i} , \widetilde{\textbf{g}_j} \right\rangle }{ \left\langle \widetilde{\textbf{g}_i} , \widetilde{\textbf{g}_i} \right\rangle } \widetilde{\textbf{g}_i} \right\rangle }{ \left\langle \widetilde{\textbf{I}_{ij}} , \widetilde{\textbf{I}_{ij}} \right\rangle - \frac{ \left\langle \widetilde{\textbf{g}_i} , \widetilde{\textbf{I}_{ij}} \right\rangle ^2 }{ \left\langle \widetilde{\textbf{g}_i} , \widetilde{\textbf{g}_i} \right\rangle } - \frac{ \left( \left\langle \widetilde{\textbf{g}_j} , \widetilde{\textbf{I}_{ij}} \right\rangle - \frac{ \left\langle \widetilde{\textbf{g}_i} , \widetilde{\textbf{g}_j}\right\rangle }{ \left\langle \widetilde{\textbf{g}_i} , \widetilde{\textbf{g}_i} \right\rangle } \left\langle \widetilde{\textbf{g}_i} , \widetilde{\textbf{I}_{ij}}\right\rangle \right) ^2 }{ \left\langle \widetilde{\textbf{g}_j} , \widetilde{\textbf{g}_j} \right\rangle - \frac{ \left\langle \widetilde{\textbf{g}_i} , \widetilde{\textbf{g}_j} \right\rangle ^2}{ \left\langle \widetilde{\textbf{g}_i} , \widetilde{\textbf{g}_i} \right\rangle } } } \end{aligned}$$

In the numerator for $\hat{\beta }_{ij}$, we note there are components that may be expressed in terms of the main effect coefficients, $\hat{\beta }_{1i}$ and $\hat{\beta }_{1j}$. These will be the following two components: $\left\langle \widetilde{\textbf{p}} , \widetilde{\textbf{g}_j} - \frac{\left\langle \widetilde{\textbf{g}_i} , \widetilde{\textbf{g}_j} \right\rangle }{ \left\langle \widetilde{\textbf{g}_i} , \widetilde{\textbf{g}_i} \right\rangle } \widetilde{\textbf{g}_i} \right\rangle = \left\langle \widetilde{\textbf{g}_j} , \widetilde{\textbf{g}_j} \right\rangle \hat{\beta }_{1j} - \left\langle \widetilde{\textbf{g}_i} , \widetilde{\textbf{g}_j} \right\rangle \hat{\beta }_{1i}$ and $\left\langle \widetilde{\textbf{p}} , \widetilde{\textbf{I}_{ij}} - \frac{ \left\langle \widetilde{\textbf{g}_i} , \widetilde{\textbf{I}_{ij}}\right\rangle }{ \left\langle \widetilde{\textbf{g}_i} , \widetilde{\textbf{g}_i} \right\rangle } \widetilde{\textbf{g}_i} \right\rangle = \left\langle \widetilde{\textbf{p}} , \widetilde{\textbf{I}_{ij}} \right\rangle - \left\langle \widetilde{\textbf{g}_i} , \widetilde{\textbf{I}_{ij}}\right\rangle \beta _{1i}$

As a result, in terms of the main effect coefficients, $\hat{\beta }_{1i}$ and $\hat{\beta }_{1j}$, we may write the pairwise epistasis interaction term as

$$\begin{aligned} \hat{\beta }_{ij} = \frac{ \left\langle \widetilde{\textbf{p}} , \widetilde{\textbf{I}_{ij}} \right\rangle - \left\langle \widetilde{\textbf{g}_i} , \widetilde{\textbf{I}_{ij}}\right\rangle \beta _{1i} - \frac{ \left\langle \widetilde{\textbf{I}_{ij}} , \widetilde{\textbf{g}_j} - \frac{ \left\langle \widetilde{\textbf{g}_i} , \widetilde{\textbf{g}_j}\right\rangle }{ \left\langle \widetilde{\textbf{g}_i} , \widetilde{\textbf{g}_i} \right\rangle } \widetilde{\textbf{g}_i} \right\rangle }{ \left\langle \widetilde{\textbf{g}_j} , \widetilde{\textbf{g}_j} \right\rangle - \frac{ \left\langle \widetilde{\textbf{g}_i} , \widetilde{\textbf{g}_j} \right\rangle ^2}{ \left\langle \widetilde{\textbf{g}_i} , \widetilde{\textbf{g}_i} \right\rangle } } \left( \left\langle \widetilde{\textbf{g}_j} , \widetilde{\textbf{g}_j} \right\rangle \hat{\beta }_{1j} - \left\langle \widetilde{\textbf{g}_i} , \widetilde{\textbf{g}_j} \right\rangle \hat{\beta }_{1i}\right) }{ \left\langle \widetilde{\textbf{I}_{ij}} , \widetilde{\textbf{I}_{ij}} \right\rangle - \frac{ \left\langle \widetilde{\textbf{g}_i} , \widetilde{\textbf{I}_{ij}} \right\rangle ^2 }{ \left\langle \widetilde{\textbf{g}_i} , \widetilde{\textbf{g}_i} \right\rangle } - \frac{ \left( \left\langle \widetilde{\textbf{g}_j} , \widetilde{\textbf{I}_{ij}} \right\rangle - \frac{ \left\langle \widetilde{\textbf{g}_i} , \widetilde{\textbf{g}_j}\right\rangle }{ \left\langle \widetilde{\textbf{g}_i} , \widetilde{\textbf{g}_i} \right\rangle } \left\langle \widetilde{\textbf{g}_i} , \widetilde{\textbf{I}_{ij}}\right\rangle \right) ^2 }{ \left\langle \widetilde{\textbf{g}_j} , \widetilde{\textbf{g}_j} \right\rangle - \frac{ \left\langle \widetilde{\textbf{g}_i} , \widetilde{\textbf{g}_j} \right\rangle ^2}{ \left\langle \widetilde{\textbf{g}_i} , \widetilde{\textbf{g}_i} \right\rangle } } } \end{aligned}$$

From this we observe that the numerator of $\hat{\beta }_{ij}$ is determined upon the numerical value of the product $\left\langle \widetilde{\textbf{p}},\widetilde{\textbf{I}_{ij}}\right\rangle$ and zero values for $\hat{\beta }_{1i}$ and $\hat{\beta }_{1j}$ do not imply $\hat{\beta }_{ij} = 0$. So insignificant interaction terms, or value of $\hat{\beta }_{ij} = 0$ does not imply $\hat{\beta }_{1i} = 0$ or $\hat{\beta }_{1j} = 0$ and on the other hand $\hat{\beta }_{1i} = 0$ or $\hat{\beta }_{1j} = 0$ does not imply $\hat{\beta }_{2i} = 0$, $\hat{\beta }_{2j} = 0$ or $\hat{\beta }_{ij} = 0$. This may be considered a warning that we may not be justified only searching main effects for pairwise epistasis. Rather a biological reason for pruning loci, such as pruning of SNPs in linkage disequilibrium (LD), for epistasis may be more justified. For example, in the rat data set we applied the epistasis algorithms to loci screened from a previous GWAS study because of biological interest in those loci for the phenotype of BMI [21–23]. In a recent study in yeast, Ang et al. observed that epistasis most often occurred between loci with minor allele frequency between five and ten percent [45], so pruning loci for epistasis based on minor allele frequency may be another strategy for pruning the search space for epistasis detection for biological reasons.

### Exclusive detection of interactions by respective interaction terms

It is not fully understood how epistatic interactions evolve, are maintained, or are structured in biological systems considering that methodologies specifically designed to systematically identify non-Cartesian interactions are not common [13–15]. As examples of the possible complexity of epistasis, Li and Reich propose over a hundred full penetrance interaction models, some of which are not linearly separable [46]. To test our methodology’s capability of supporting multiple interaction models when detecting epistasis, we select the exclusive-or (XOR) penetrance model (model M170 in Li and Reich) in addition to the Cartesian product (S2 File). We choose XOR because of its extreme difference compared to the standard Cartesian model in that the phenotype is entirely dependent on the MLG. Therefore, assuming full penetrance, XOR is not linearly separable or detectable using any single-locus analyses like GWAS. Due to these aspects, the XOR model is commonly considered to not be biologically plausible, with some noted exceptions [47, 48].

Before investigating if unique instances of epistasis can be detected in real-world systems using Cartesian and XOR models, we test if Cartesian and XOR interaction terms detect interactions that follow their respective models with higher fidelity than the alternate model in simulated data. To do this, we simulate nine pair-wise interactions under both models using 18 genetic loci from the rat GWAS data [21–23], generating two datasets - one with Cartesian interactions and one with XOR interactions. We generate the interactions using a previously published method [49] in which the BMI phenotype is ranked from lowest to highest magnitude and a portion of the observations are permuted to build interactions using the respective interaction model. Each SNP in the dataset is assigned to be in a pair-wise interaction with the adjacent SNP (i.e., SNP1 is interacting with SNP2, SNP3 is interacting with SNP 4, and so on and so forth). We use our algorithm with both Cartesian and XOR interaction terms and compare the assessment of significance of all possible 18 choose 2 (153) pair-wise interactions in both datasets in Python v. 3.11. We also develop a standard regression method that assumes a Cartesian interaction term by default (representing available standard approaches) to determine if the results match those of our model with a Cartesian interaction term.

### Application to body mass index data

We have applied our algorithm to a dataset of an outbred, related rat (*Rattus norvegicus*) population of males and females derived from eight inbred founders (Heterogenous Stock, [50]) in order to detect possible epistatic interactions associated with Body Mass Index (BMI) as an exploratory analysis for our algorithm. We also use the rat data to showcase the extension of our algorithm to higher order epistasis by investigating three-way interactions between putative Quantitative Trait Loci (QTLs) identified via GWAS [21–23]. Additionally, we have applied our algorithm to a dataset of an inbred population of mice (*Mus musculus*) derived from 17 mouse strains [24, 25] to compare epistatic interactions, both Cartesian and XOR, in a closely related species to rat.

### Assessing computational efficiency

To calculate the relative speed our algorithm compared to a standard linear regression method, we use the same regression method we constructed in the term-specific interaction test experiment. We sample from the GWAS data [21–23] to generate thirteen datasets of increasing sample size (10, 100, 1,000, 2,000, 3,000, 4,000, 5,000, 6,000, 7,000, 8,000, 9,000, and 10,000) and use the BMI phenotype as the response variable. For sample sizes greater than 5,000, we copy rows of the existing data to generate datasets of larger dimension. We also generate three datasets containing 10, 100, and 1,000 SNPs with a constant sample size (5,566) to determine if the algorithm gains efficiency as SNP number increases. We run all algorithms to completion (assess all possible pairwise comparisons) in Python v. 3.11 and measure the computation time of both methods (assuming the Cartesian interaction term) using standard Python libraries on two systems: a Macbook Pro®with the M1 Pro®chip architecture (3.2GHz) and a PC with an Intel ®Xeon®Silver 4201R CPU (2.40GHz) and an NVIDIA®RTX A2000 dedicated GPU. For the PC test, we use CuPY to additionally test GPU computation with our algorithm. We perform 10 replicate runs for each dataset and calculate the average time as well as summary statistics (means, variances, standard errors, F-tests, and T-tests). The average standard regression time is divided by our algorithm’s time to obtain comparative speed ratios.

### Detecting epistasis

The GWAS dataset of BMI residuals (corrected for sex and location) in 5,566 rats containing approximately 129,000 autosomal SNPs is used for our rat analysis [21–23]. To reduce the dimensionality of the dataset for exploratory purposes, we select the top 10,000 SNPs (SNPs with the 10,000 lowest *p*-values) from the original GWAS for further analysis because of their biological interest and relevance and to compare findings to the previous GWAS and studies investigating obesity-related traits. For mice, we use the genotype/phenotype information from the Wellcome Trust Mouse Genomes Project [24, 25] found in the BGLR package [51] in R [52]. We did not prune this dataset because it is close in dimensionality ( 10,000 SNPs) to the pruned rat dataset. In total, we extract the genotype and BMI phenotype data available for 10,347 SNPs for 1,814 mice.

To account for the relatedness of the samples and population structures, the genetic relatedness matrices (GRMs) are calculated for the rat data using the method of Yang et al. using the GCTA software tool [53] and the mouse data using the method of Sul et al. [54]. We use the GRMs to calculate the variance component analysis for the phenotype of BMI for each data set using the methods of Joo et al. [55] and Kang et al. [56, 57] as implemented in the mmer function with method EMMA of the sommer package version 3.2 in R [58]. From the variance component analyses, we obtain the inverses of the covariance matrices for both data sets as returned by the sommer package and then use the the *sqrtm* function from the expm package version 0.999-7 in R to obtain their square roots [59]. These inverse square root covariance matrices are then used to correct for population structure and essentially solve a generalized linear model by performing a weighted least squares by multiplying each variable in the model by the half inverse matrix before applying our algorithms as a pre-processing step. This “mixed model trick” is summarized by Suh et al. [54].

Both Algorithms, 1 and 2, are implemented in Python v. 3.9 for our experiments in detecting epistasis under Cartesian and XOR interaction models. To calculate the *p*-values for the two-sided T tests for the test statistics returned from our algorithms, the t.sf function from the scipy.stats package is used [60]. For two-way epistasis, Algorithm 1 is applied to all possible pairs of 10,000 SNPs in the rat data set and all possible pairs of 10,347 SNPs in the mouse data set. This is done as two separate experiments for each data set, once for the XOR model and another for the Cartesian (product) model. In addition, for the XOR model for all 10,000 SNPs for the rat data set, *p*-values are also calculated using the permutation testing algorithm with 1,000 permutations. (This was computationally intensive since we calculate nearly fifty million tests per permutation and was done in parallel.) To account for multiple testing, FDR is implemented in the *fdrcorrection* function in the statsmodels package in Python [61] using a *p*-value threshold of 0.05. For three-way epistasis in the rat data set, we apply Algorithm 2 to the 18 putative main effect QTLs from the rat GWAS study [21–23] to determine if any are involved in significant three-way epistatic interactions.

### Pruning redundant epistatic events

For mice, we map all SNPs to their respective genomic location (assembly GRCm39) and retain those that map to the 19 autosomes. This leaves us with 9,525 SNPs for further analyses. Although GWAS SNPs were LD-pruned in rats, it is likely that many detected pairs are redundant in that one or both epistatic partners of a particular pair are in LD with others in close genomic proximity. In fact, many epistatic pairs where in close proximity (within 10 to 100 base pairs (bp)) to one another in both systems (S1File, S3File). We choose a conservative threshold of 10 megabases (Mb) upstream or downstream to prune redundant pairs in both species. All pruning steps are performed in R [52]. Under both Cartesian and XOR models, to prune interchromosomal pairs, four conditions must be met when comparing epistatic pairs: 1.) locus one in pair one and locus one in pair two are on the same chromosome, 2.) locus two in pair one and locus two in pair two are on the same chromosome, 3.) the absolute value of the difference in chromosomal position (in bp) between locus one in pair one and locus one in pair two is less than 10Mb, and 4.) the absolute value of the difference in chromosomal position between locus two in pair one and locus two in pair two is less than 10Mb. If all four conditions are true, the epistatic pair with the lower FDR-corrected *p*-value for our epistasis test is retained while the other is omitted. We then check for mirror redundancies where two pairs technically meet the above criteria, but the chromosomal combination is reversed. The pair that is identified first is retained while the other is omitted. To prune intrachromosomal pairs, we check if two pairs exist on the same chromosome and then use the same bp criteria as outlined above for to prune pairs. We also take an additional pruning step for intrachromosomal pairs where the absolute value of the bp difference between locus one and locus two is less than 10Mb. If this condition is met, the pair is omitted. This step removes close-acting cis-regulatory epistatic events as a byproduct of controlling for high LD in these model systems. We perform this pruning strategy to highlight epistatic hubs in both genomes and only consider the most significant sources of epistasis in this exploratory study.

There are nine total MLGs possible under pair-wise epistasis of two biallelic SNPs (S2 File). We limit our results to only those pairs where all nine MLGs occur. Additionally, we remove any significant epistatic pair, both in Cartesian and XOR, that do not have at least 10 or 5 observations of all nine MLGs in the datasets in rats and mice, respectively. Under Hardy-Weinberg assumptions, if the minor allele frequencies in two loci are 0.10, then we only expect to observe a genotype frequency for a double minor homozygote of 0.0001. We select the cutoff observations of 10 and 5 in rats and mice, respectively to ensure the combinatory genotype frequencies of the MLGs to be sufficiently above what would be expected if minor allele frequencies are 0.10 in both epistatic loci under Hardy-Weinberg assumptions.

### Identifying QTL-associated and non-QTL-associated epistatic events and epistatic hubs

After our initial pruning steps, we determine if a locus was associated with a putative QTL for BMI (“BMI with tail” in rats and “BMI” in mice) from the original GWAS studies [21–25] to observe if most epistatic events occur at or near loci with large main effects. To accomplish this, we use lists of the putative single locus QTL from the original GWAS studies and their respective genomic locations. We record if either locus 1 or locus 2 of a pair is within 10Mb upstream or downstream of a putative QTL to count the number of epistatic pairs with one putative GWAS QTL involved. We also note if both loci of a pair are associated with a GWAS QTL to record QTL to QTL epistatic interactions. If a locus is within 10Mb upstream or downstream of a GWAS QTL, its identification is replaced by the putative QTL’s identification. We also count how many epistatic events involve each GWAS QTL. If non-QTL-associated loci are within 10Mb upstream or downstream of each other on the same chromosome, the average genomic location is calculated and all non-QTL epistatic loci’s bp positions used to calculate the average are replaced by the average bp location. This procedure identifies non-QTL-associated epistatic loci and hubs. For our analyses, any locus with 10 or more epistatic interactions are defined as an epistatic hub (GWAS QTLs included).

Under XOR, we also identify loci that are within 10Mb upstream or downstream of a GWAS QTL and replace their identifications with the respective GWAS QTL. For non-QTL-associated XOR loci, we determine which loci are within 10Mb upstream or downstream of the non-QTL-associated loci identified under the Cartesian model and replace their identification with the identification of the Cartesian locus. This allows us to determine how many epistatic loci/hubs are shared between both models. For loci that are unique to the XOR model, we apply the same procedure used to determine non-QTL-associated loci under Cartesian model where we calculate the average genomic location among loci if on the same chromosome and within 10Mb upstream or downstream of each other. Thus, we identify loci specific to each interaction model as well as loci shared between models.

### Identifying potential phantom epistasis

Phantom epistasis, the phenomenon where significant statistical epistatic events are detected between large additive effect loci in strong LD with interacting loci, can occur and has been detected [62]. Although our pruning strategy controls for *cis* LD and the zero or small independent effects of each locus expected under the XOR model partially control for strong additive effects between two loci [46], *trans* LD can occur across vast genomic distances due to forces including, but not limited to, selection and inbreeding [63]. To investigate if phantom epistasis is occurring in our rat data, we calculate LD statistics (D’ and R^2^) for all pairwise epistatic interactions involving the putative GWAS QTL with the largest additive effect (chr1.281788173_G) under both Cartesian and XOR models. For this analysis, we use the *LD* function in the R package, genetics [64].

### Gene set enrichment and Kegg pathway analysis

To perform functional annotation for loci involved in epistatic events, we query protein-coding gene models, non-protein coding gene models, and pseudogene models from the Rat Genome Database (https://rgd.mcw.edu/) for rats (assembly Rnor 6.0) and mice (assembly GRCm39). We retrieve any model that is 1Mb upstream or downstream from each epistatic locus/hub. We then convert the species-specific gene model symbols from the respective database to Entrez IDs. Any returned queries that do not have associated Entrez IDs are omitted. We use this list to identify the enrichment of GO (gene ontology) terms (cellular component, molecular function, and biological process) using the BioConductor [65] package clusterProfiler [66, 67] in R with FDR correction (*p*-value cutoff$= 0.01$; *q*-value cutoff$= 0.05$). We also perform Kyoto Encyclopedia of Genes and Genomes (KEGG) pathway enrichment analysis using clusterpPofiler (*p*-value cutoff$= 0.05$; *q*-value cutoff$= 0.02$). Dotplot figures of enriched ontological terms are made using the DOSE [68] package in R.

### Identifying three-way epistatic interactions between putative QTLs

To test the extension of our algorithm for identifying higher order epistasis, we explore three-way epistatic interactions among the 18 putative QTL identified in the original rat GWAS [21–23] under both Cartesian and XOR interaction models. Triplets that have an associated experimental *p*-value < 0.05 are retained and we record occurrences of three-way epistasis for each GWAS QTL. In addition, the triplets between each interaction model are compared for overlap. Since all significant triplets in both experiments are unique in terms of genomic location and are between putative QTLs, no pruning or epistatic locus identifications are performed for this analysis.

## Results

### Interaction terms excel with interactions simulated assuming their respective model

After FDR correction, pairwise interactions simulated with the Cartesian model are detected better with the Cartesian interaction term compared to the XOR interaction term. The average corrected *p*-value of the nine simulated Cartesian interactions with the Cartesian interaction term is 1.86E-06 compared to 0.18 with XOR (S1 File). The XOR interaction term does, however, identify five of the nine Cartesian interactions as significant. With stricter FDR correction (i.e., a larger number of SNPs), most of these, if not all, would likely not remain significant as they are marginal. As for XOR simulated interactions, after FDR correction, none of the possible 153 pairwise comparisons are significant using the Cartesian interaction term (S1 File). However, using our algorithm with an XOR interaction term, the nine simulated XOR interactions have the lowest adjusted *p*-values by a large margin compared to any of the other possible 144 interactions (average *p*-value of XOR interactions = 6.31E-26). The non-adjusted and adjusted *p*-values from the standard regression approach with the Cartesian interaction term match exactly to the non-adjusted and adjusted *p*-values derived from our algorithm with the Cartesian term (S1 File).

### Epistasis algorithm efficiency scales with sample size

In both the Mac and PC tests, our algorithm outperforms the standard regression approach as sample size increases (S1 File). Faster computation times are reached quickly on the Mac, at a sample size of 3,000 (1.9X speed up), and speed increases of approximately 2.3X are achieved at samples sizes 6,000 and above. On the PC, speed increase does not occur until a sample size of 5,000 for the GPU and a sample size of 6,000 for the CPU. Max speed increases are observed at a sample size of 10,000 with 1.2X for the CPU and 1.4X for the GPU. SNP number does not affect the speed ratio between algorithms as much as sample size (S1 File). With a constant sample size of 5,566, as SNPs increase from 10 to 1,000, the average speed increase is 2.4X (min = 2.1X, max = 2.6X) for the Mac system and, on the PC, average speed increases are 1.04X (min = 0.96X, max = 1.1X) and 1.1X (min = 0.97, max = 1.2X) for the CPU and GPU, respectively. These results illustrate that our epistasis algorithm scales well with sample size and will reach higher levels of efficiency with large experimental designs. However, this efficiency is system and specification dependent. GPU integration, including the integrated M1 Pro®GPU, yields faster computation speeds.

### Most epistatic interactions and hubs occur at non-QTL-associated loci

**Fig. 1:**
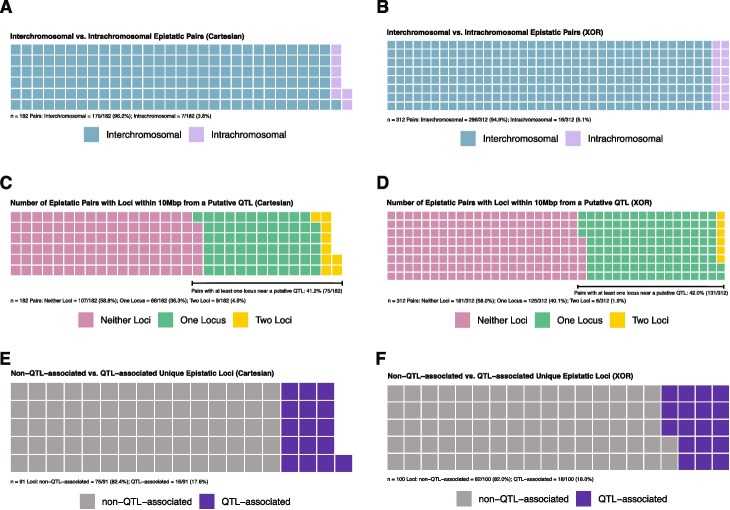
Proportions of epistasis detected in rats. **A** and **B** Interchromosomal (blue squares) vs intrachromosomal (light purple squares) pairs under Cartesian (**A**) and XOR (**B**) models. **C** and **D** Number of epistatic pairs involving no (pink squares), one (green squares), or two (yellow squares) putative GWAS QTL under Cartesian (**C**) and XOR (**D**) models. **E** and **F**: Non-QTL-associated (gray squares) and QTL-associated (dark purple squares) epistatic loci under Cartesian (**E**) and XOR (**F**) models

For the Cartesian experiment in rats, our method detects 4,158 (86.8%) interchromosomal and 634 (13.2%) intrachromosomal significant pairs (4,792 total; S1 File). After MLG pruning, this reduces to 3,109 (90.4%) interchromosomal and 329 (9.6%) intrachromosomal pairs (3,438 total; S1 File). After redundancy pruning, this is further reduced to 175 interchromosomal pairs (96.2%) and seven intrachromosomal pairs (3.8%) (182 total) (Fig. 1A; S1 File). There are 182 Cartesian pairs after all pruning. Of these, there are 66 pairs (36.3%) containing one QTL-associated locus and 9 (4.9%) QTL-QTL interactions (Fig. 1C; S1 File). However, most pairs are between two non-QTL-associated loci (107 pairs; 58.8%). There are a total of 91 Cartesian epistatic loci. Of these, 75 are non-QTL-associated (82.4%) (Fig. 1E; S1 File). Of the 18 putative GWAS QTL, chr7:8599340_A, chr18.32316331_A, and chr5:72916242_T are not detected as epistatic loci (S1 File). It is important to note that chr18.32316331_A is physically close to another putative QTL, chr18.27348077_G (4,968,254 bp apart), and is not represented as loci we detect are physically closer to chr18.27348077_G.

In mice, 81 epistatic pairs are detected under the Cartesian model. However, 65 of these involve at least one locus on the X chromosome and are omitted as we are only investigating autosomal loci, leaving 16 pairs. None of these pairs are intrachromosomal (S3 File). After MLG pruning, eight pairs remain and after redundancy pruning only one pair remains between chr3.44666611, and chr12.7079769 (S3 File). In rats, the XOR model yields more significant pairs compared to Cartesian (31,182 vs. 4,792) (S1 File). Of the 31,182 significant pairs, 27,774 are interchromosomal (89.1%) and 3,408 are intrachromosomal (10.9%; S1 File). After MLG pruning, this is reduced to 11,016 (86.4%) interchromosomal pairs and 1733 (13.6%) intrachromosomal pairs (12,749 pairs total; S1 File). After redundancy pruning, this is further reduced to 296 (94.9%) interchromosomal pairs and 16 (5.1%) intrachromosomal pairs (312 pairs total) (Fig. 1B; S1 File). Most pairs (181 (58.0%)) are between two non-QTL-associated loci while there are 125 (40.1%) pairs involving one QTL-associated locus and six (1.9%) QTL-QTL interactions (Fig. 1D; S1 File). Epistatic pairs involve 100 loci where 82 (82.0%) are non-QTL-associated (Fig. 1F; S1 File). All 18 putative GWAS QTL are represented under the XOR model.

**Fig. 2:**
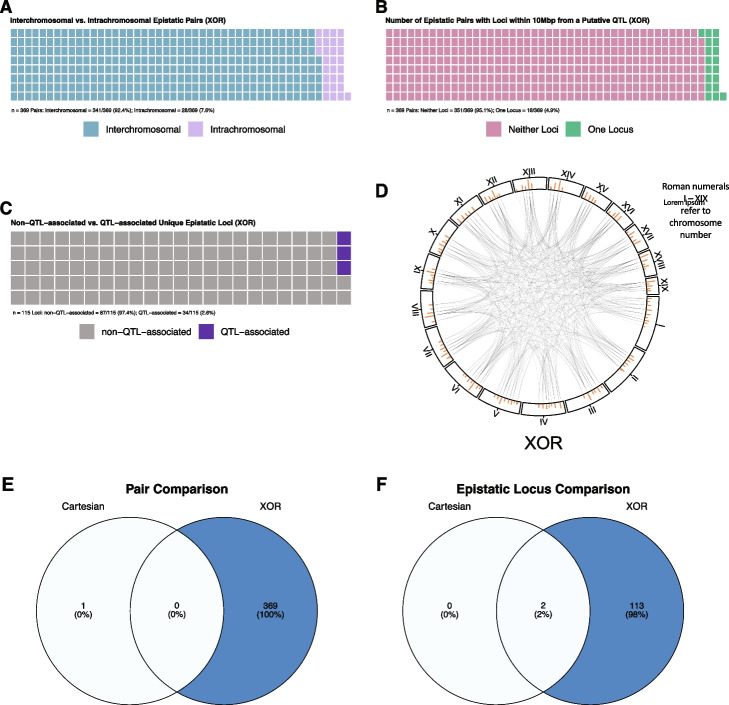
Representations of epistasis in mice. **A** Interchromosomal (blue squares) vs. intrachromosomal (light purple squares) pairs under XOR model. **B** Number of pairs involving no (pink squares), one (green squares), or two (yellow squares) putative GWAS QTL under XOR model. **C** Non-QTL-associated (gray squares) and QTL-associated (dark purple squares) epistatic loci under XOR model. **D** Interaction plot of XOR epistasis across mouse autosomes. Autosome numbers are depicted as roman numerals and increase clockwise. Orange bars represent counts of epistatic instances per locus. **E** Venn diagram of epistatic pairs under Cartesian (left circle) model, the intersect between models (center), and under XOR (right circle) model. **F** Venn diagram of epistatic loci under Cartesian (left circle) model, the intersect between models (center), and under XOR (right circle) model. **E** and **F** Color gradient illustrates low (white) to high (blue) occurrences

In mice, under the XOR model, there are 468,596 significant pairs. After we map these loci to genomic locations and remove any pair involving an non-autosomal locus, 25,055 pairs remain with 23,688 (94.5%) interchromosomal and 1,367 (5.5%) intrachromosomal (S3 File). After MLG pruning, this is reduced to 13,328 (95.0%) interchromosomal pairs and 707 (5.0%) intrachromosomal pairs (14,035 pairs total; S3 File). Finally, after redundancy pruning, this is reduced to 341 (92.4%) interchromosomal pairs and 28 (7.6%) intrachromosomal pairs (369 pairs total) (Fig. 2A; S3 File). As in rats, and due to there only being 3 putative GWAS QTL for BMI in mice we are able to map to a genomic location, most pairs (351 (95.1%)) are between two non-QTL-associated loci while there are 18 (4.9%) pairs containing one QTL-associated locus (Fig. 2B; S1 File). There are no QTL-QTL interactions detected in mice. In mice, XOR yields 115 unique epistatic loci where 112 (97.4%) are non-QTL-associated.

**Fig. 3:**
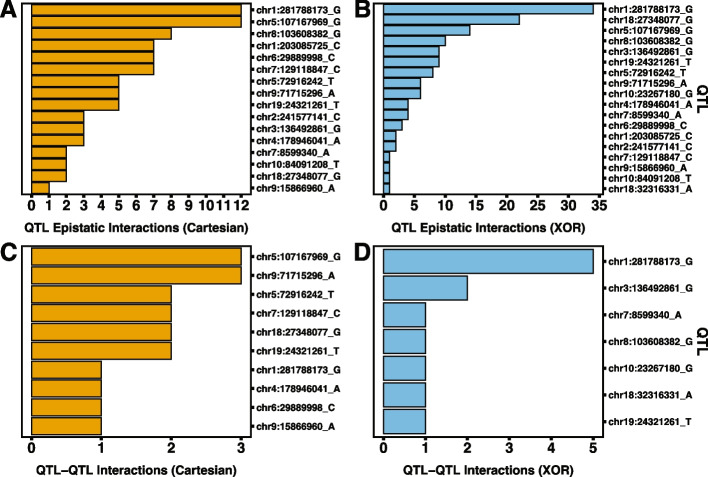
Bar graphs of occurrences of QTL-associated epistasis in rats. **A** and **B** Bar graphs of instances of epistatic events for GWAS putative QTL under Cartesian (**A**, orange bars) and XOR (**B**, blue bars) models. **C** and **D** Bar graphs of instances of QTL to QTL epistatic events for GWAS putative QTL under Cartesian (**C**, orange bars) and XOR (**D**, blue bars) models

It is important to note that a large amount of significant epistatic pairs involve non-autosomal or unmappable loci in mice. In future analyses, we aim to explore interactions that involve non-autosomal loci within the mouse cohort and in other species. The rank order and number of interactions per GWAS QTL differ between experiments and across species. In rats, under the Cartesian model, the largest QTL-associated hubs are chr1.281788173_G and chr5.107167969_G with 12 interactions each (Fig. 3A; S1 File). chr1.281788173_G is also the locus with the largest main effect signal in the rat GWAS (S1 File). Under the XOR model, there are four QTL-associated hubs identified (Fig. 3B; S1 File). As in Cartesian, chr1.281788173_G is the largest QTL-associated hub with 34 interactions. This is followed by chr18:27348077_G with 25 interactions. XOR QTL-associated hubs also include chr5:107167969_G and chr8:103608382_G. Under the Cartesian model, 10 of the putative GWAS QTLs are in QTL-QTL interactions (Fig. 3C; S1 File). Two putative GWAS QTLs are in three QTL-QTL interactions while four are in two QTL-QTL interactions. The remaining four are in one. The Cartesian QTL hubs, chr5.107167969_G and chr1.281788173_G are involved in QTL-QTL interactions, but there doesn’t seem to be a clear relationship between number of epistatic interactions and number of QTL-QTL interactions. Under the XOR model, seven putative GWAS QTL are in QTL-QTL interactions (Fig. 3D; S1 File). However, the rank orders and QTL representations are distinct between models. For example, under XOR, chr1.281788173_G has the largest occurrences of QTL-QTL interactions with five. Under XOR, there also is not a clear relationship between the number of QTL epistatic events a hub is involved in and the propensity of QTL-QTL interactions.

**Fig. 4:**
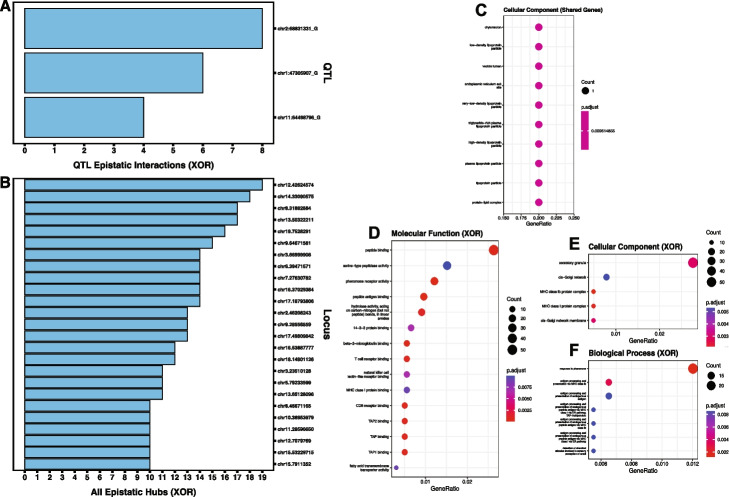
Bar graphs of epistatic interactions and dot plots of GO term gene set enrichment in mice under the XOR model. **A** Bar graph of instances of QTL-associated epistatic events for GWAS putative QTL under XOR model. **B** Bar graph of instances of epistasis for all epistatic hubs (QTL-associated and non-QTL-associated) under XOR model. **C** dot plots for enriched cellular component GO terms. **D** through **F** dot plots for enriched GO terms for biological processes (**D**), cellular components (**E**), and molecular functions (**F**) for XOR-specific epistatic loci. **C** through **E** size of dot corresponds to the number of genes associated with that enrichment. Color gradient illustrates level of significance with higher *p*-values in blue and lower *p*-values in red

In mice, under the Cartesian model, QTL and QTL-QTL interactions do not occur as only one significant pair, involving two non-QTL-associated loci, is detected. However, under the XOR model, all three putative QTL are involved in a least one epistatic interaction; however none are hubs (Fig. 4A; S3 File). The largest QTL epistatic loci is chr2.68831331_G with eight interactions. No QTL-QTL interactions are detected in mice under the XOR model.

**Fig. 5:**
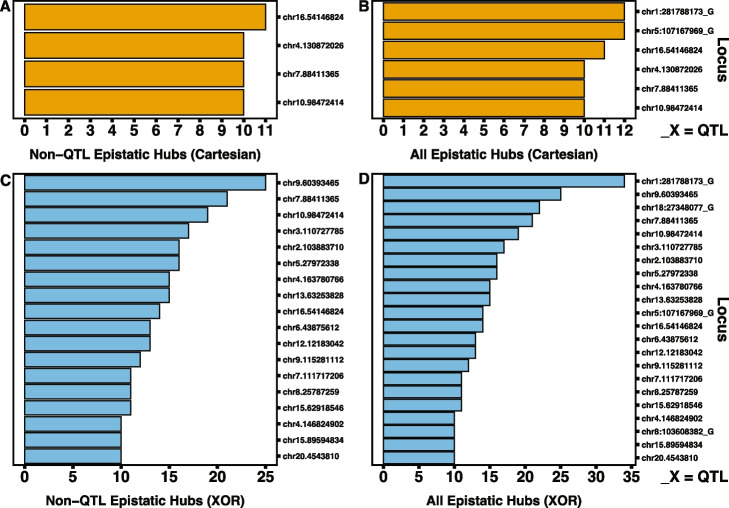
Bar graphs of epistatic events in non-QTL-associated hubs and all hubs in rats. **A** and **B** Bar graphs of instances of epistatic events for non-QTL-associated hubs (**A**) and all hubs (**B**) under Cartesian model (orange bars). **C** and **D** Bar graphs of instances of epistatic events for non-QTL-associated hubs (**C**) and all hubs (**D**) under XOR model (blue) bars). **B** and **D** Loci on y axes ending with “_” and allele designation are GWAS putative QTL

In rats, there are four Cartesian non-QTL-associated epistatic hubs and 18 under XOR (Fig. 5A,C; S1 File). The largest non-QTL-associated epistatic hubs are chr16.54146824 under Cartesian and chr9.60993465_G under XOR. As we observe with GWAS QTL epistasis, the rank order and hub sizes of non-QTL-associated hubs differ between experiments (Fig. 5A,C; S1 File). When including putative GWAS QTLs as hubs, there are a total of six epistatic hubs identified under the Cartesian model (Fig. 5B; S1 File) and 22 under the XOR model (Fig. 5D; S1 File). GWAS QTLs account for 33% (2/6) and 18.2% (4/22) of Cartesian and XOR hubs, respectively. Despite the rank order of hubs being largely distinct between experiments, the largest hub under both Cartesian and XOR models is the putative GWAS QTL with the largest main effect signal in the rat GWAS [21–23], chr1.281788173_G. In mice, under the XOR model, all 25 hubs are non-QTL-associated. The largest is chr12.42624574 with 19 interactions (Fig. 4B, S3 File).

In rats, there is a significant correlation between the significance of SNPs in the GWAS considering its *p*-value (i.e. -log10*p*-value) and the number of epistatic interactions under the XOR model when considering all epistatic SNPs (*p* = 0.0112, r = 0.253; S2 File) and only QTL-associated SNPs (*p* = 0.00249, r = 0.667; S2 File). This relationship does not occur under the Cartesian model. We did not perform this analysis in mice as we did not have access to the GWAS statistics for all SNPs. Phantom epistasis detection results illustrate that no measurements of pairwise LD (D’ and R^2^), for epistatic pairs involving chr1.281788173_G, either in Cartesian or XOR, suggest strong association between loci (S1 File).

Permutation test results in rats under the XOR model yielded 29 XOR specific loci compared to 25 when just using FDR correction alone. Of the 29 XOR specific loci after 1,000 permutations, most (23) overlap (are within 1 Mb of a similar locus with FDR correction alone) while six are only found after 1,000 permutations (S1 File). In this instance, permutation testing leads to the detection of additional epistatic loci. We only apply the permutations algorithm to epistatic pairs from rat data under the XOR model to both test the algorithm’s capability and to verify detection of XOR statistical epistasis in a living system.

### Cartesian and XOR share common epistatic loci while epistatic landscapes are distinct

**Fig. 6:**
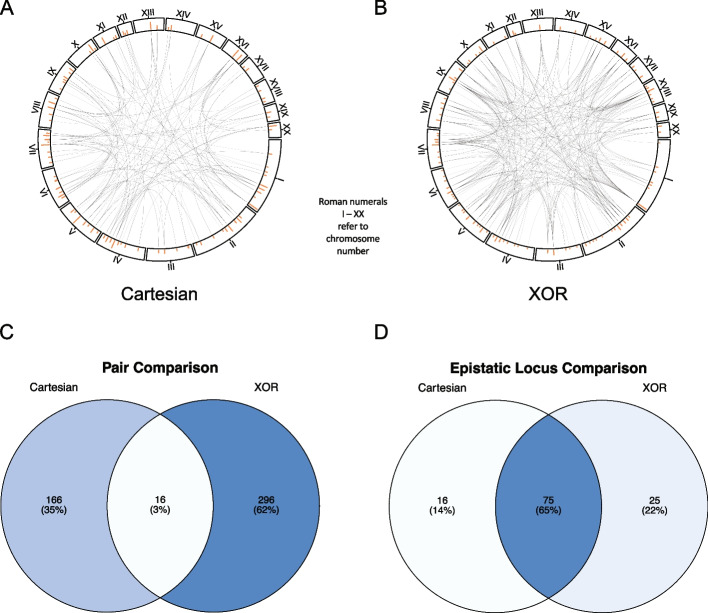
Interaction plots and epistatic model comparisons in rats. **A** and **B** Interaction plots of Cartesian (**A**) XOR (**B**) epistasis across rat autosomes. Autosome numbers are depicted as roman numerals and increase clockwise. Orange bars represent counts of epistatic instances per locus. **C** Venn diagram of epistatic pairs under Cartesian (left circle) model, the intersect between models (center) and under XOR (right circle) model. **D** Venn diagram of epistatic loci under Cartesian (left circle) model, the intersect between models (center) and under XOR (right circle) model. **C** and **D** Color gradient illustrates low (white) to high (blue) occurrences

In rats, the interaction landscapes of Cartesian and XOR two-way epistasis are mostly distinct (Fig. 6A,B; S1 File). Out of the 182 Cartesian and 312 XOR pairs, only 16 pairs (3%) reach significance under both models (Fig. 6C; S1 File). However, of the 91 and 100 epistatic loci that occur under Cartesian and XOR models, respectively, 75 (65%) are shared Fig. 6D; S1 File). Although most epistatic loci are shared between Cartesian and XOR, distinct significant two-way epistatic interactions occur under each model.

In mice, only one epistatic pair is significant and is distinct to the Cartesian model (i.e., does not reach significance under the XOR model) (Fig. 2E; S3 File). Only two epistatic loci are significant under the Cartesian model. These two loci also reach significance under the XOR model (Fig. 2F; S3 File).

### Enriched terms and pathways associated with metabolism detected from epistatic loci

**Fig. 7:**
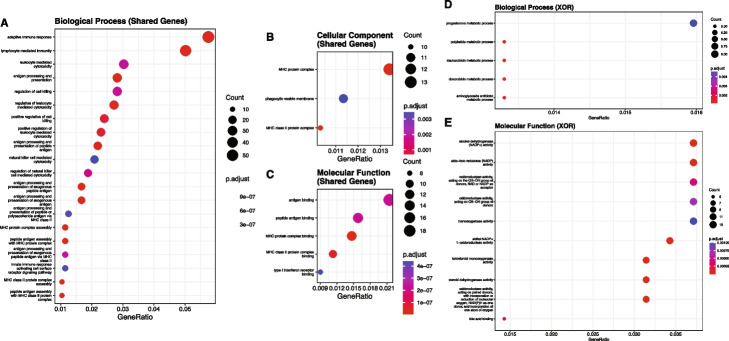
Dotplots of gene set enrichment in rats. **A** through **C** dot plots for enriched GO terms for biological processes (**A**), cellular components (**B**), and molecular functions (**C**) for shared genes between Cartesian and XOR models. **D** and **E** dot plots for enriched GO terms for biological processes (**D**) and molecular functions (**E**) for XOR-specific epistatic loci. **A** through **E** size of dot corresponds to the number of genes associated with that enrichment. Color gradient illustrates level of significance with higher *p*-values in blue and lower *p*-values in red

In rats, the 16 Cartesian-unique and 25 XOR-unique epistatic loci are analyzed using gene set enrichment. Additionally, the 75 loci that are shared between the models and the 18 putative QTLs are also analyzed. Under the Cartesian model, the 16 Cartesian-unique loci led to no significant enrichment of cellular component, biological process, or molecular function. However, one KEGG pathway was enriched - Ribosome (S1 File). Enrichment of the 75 shared loci reveal biological processes, cellular components, and molecular functions associated largely with immunity (Fig. 7A-C; S2 File). Under the XOR model, five enriched biological processes are identified, all of which are metabolic in nature (Fig. 7D; S1 File; S2 File). A total of ten molecular functions are enriched with the vast majority being oxidoreductase activities and carboxylic acid binding (Fig. 7E; S1 File; S2 File). No cellular components are significantly enriched. A total of 17 KEGG pathways are enriched. Notable metabolism-associated KEGG pathways are nitrogen metabolism, gastric acid secretion, and glucagon signaling pathway (S1 File). In mice, the two shared loci between models reveal significant enrichments of metabolic terms (Fig. 4C; S3 File). The enrichments from the 113 XOR-specific loci are mostly involved in immunity (Fig. 4D,E; S3 File). There are a total of eight biological functions, five cellular components, 15 molecular functions, and two KEGG pathways significantly enriched from XOR-specific loci. However, “detection of chemical stimulus involved in sensory perception of smell”, “fatty acid transmembrane transporter activity”, and “hydrolase activity, acting on carbon-nitrogen (but not peptide) bonds, in linear amides” are enriched metabolically-relevant processes and functions. Additionally, both KEGG pathways are metabolic in nature: "Endocrine and other factor-regulated calcium reabsorption" and "Nicotinate and nicotinamide metabolism" (S3 File).

In rats, GO term enrichments for the 75 shared epistatic loci between models yielded 75 significantly enriched biological processes, three cellular components, five molecular functions, and 46 KEGG pathways. The vast majority of these terms and pathways are involved in immunity (S1 File). In mice, the two shared epistatic loci between Cartesian and XOR models significantly enriched 10 cellular components and four KEGG pathways, all of which are involved in metabolism (S3 File). This is likely due to the gene *ApoB* being located in close proximity to the chr12.7079769 epistatic locus. Interestingly, SNPs near this gene were not implicated in the original GWAS study ([24, 25]; S3 File). In rats, the 18 putative GWAS QTL yield 14 significantly enriched biological processes and one molecular function (S1 File). Outside of “cellular response to alcohol”, all other enriched biological processes are related to immunity. The lone enriched molecular function is “type I interferon receptor binding”. The 18 putative GWAS QTL yield KEGG pathway enrichment mostly associated with immunity. However, metabolic-related pathways like “alcoholic liver disease” and “lipid and atherosclerosis” are also enriched (S1 File). In mice, the three putative GWAS QTL are enriched for one cellular component (“inner dynein arm”) and two metabolic KEGG pathways: “Glycoaminoglycan biosynthesis” and “Cholesterol metabolism” (S3 File).

### Three-way epistatic landscapes for QTL are distinct between models

**Fig. 8:**
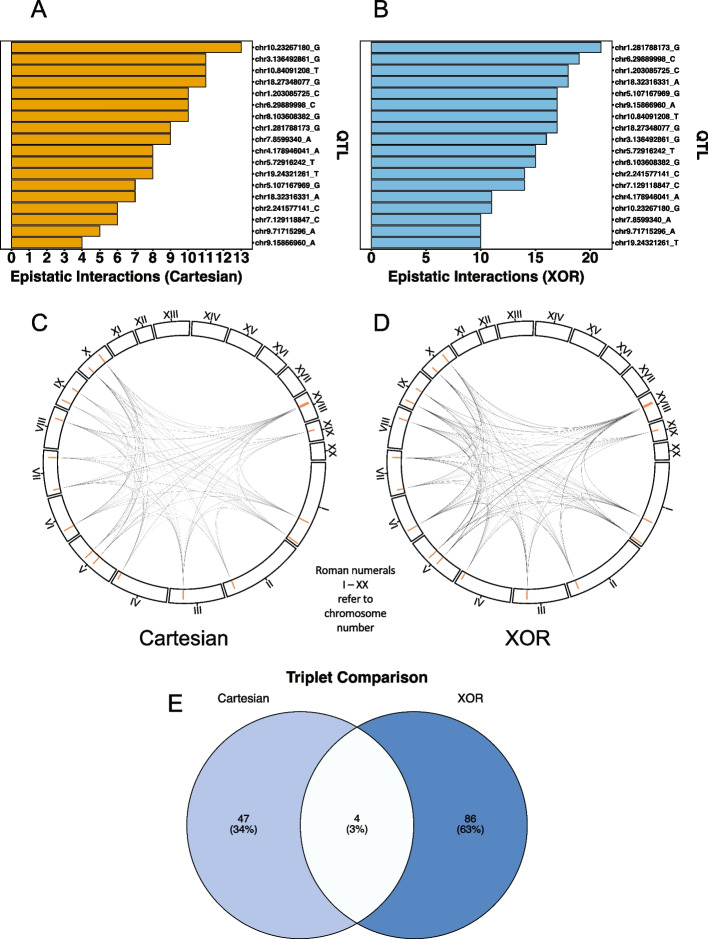
Representations of three-way epistasis in rats. **A** and **B** Bar graphs of occurrences of three-way epistatic events in GWAS QTLs under Cartesian (**A**, orange bars) and XOR (**B**, blue bars) models. **C** and **D** Interaction plots of Cartesian (**C**) XOR (**D**) three-way epistasis of GWAS QTLs across rat autosomes. Autosome numbers are depicted as roman numerals and increase clockwise. Orange bars represent counts of epistatic instances per locus. **E** Venn diagram of epistatic triplets for GWAS QTLs under Cartesian (left circle) model, the intersect between models (center) and under XOR (right circle) model. **E** Color gradient illustrates low (white) to high (blue) occurrences

Using the XOR model, significant three-way epistatic interactions between putative GWAS QTL result in more and larger epistatic hubs compared to the Cartesian model (seven GWAS QTL hubs in Cartesian vs. all 18 GWAS QTLs in XOR) (Fig. 8A,B; S1 File). Additionally, as we also observe in the two-way experiments, the epistatic landscapes and rank order of epistatic loci are distinct between the two models (Fig. 8A-E; S1 File). Furthermore, out of the 51 significant epistatic triplets in Cartesian and 90 in XOR, only 4 triplets are shared between them despite sharing all epistatic loci (the 18 putative GWAS QTLs) (Fig. 8E; S1 File). A similar result occurred in the two-way experiment (Fig. 6D; S1 File). There is no significant correlation between the absolute value of the GWAS betas or the GWAS -log10*p*-values and the number of three-way interactions for the 18 putative GWAS QTL under either interaction model (S2 File).

## Discussion

### Epistasis occurs widely at non-QTL-associated locations

Our findings point to the potential ubiquity of epistasis in living systems as we detect numerous epistatic pairs and loci under two distinct interaction models with many of them unique to either Cartesian or XOR. Furthermore, most interactions detected occur at loci not associated with a GWAS QTL (more than 10Mb upstream or downstream) in both species. However, in rats, the largest epistatic hub under both Cartesian and XOR models is chr1.281788173_G, which is also the putative QTL with the largest signal found in the rat GWAS study. In mice, under the XOR model, all epistatic hubs are non-QTL-associated. One explanation for this is that there are only three putative QTL for BMI from the mouse GWAS study that are mapped to a genomic location. Two studies investigating genome-wide non-additive effects in yeast found results to the contrary where a strong positive correlation between the main effect size of a locus and the number of interactions it was involved in is observed [7, 8]. Although we observe strong correlations in the two-way experiment between the GWAS *p*-value of a locus and number of interactions under the XOR model in rats, it is important to consider that most hubs, both under Cartesian and XOR models and in both species, are not located near a putative QTL and hence did not have strong main effects in the original GWAS study.

There are several possible explanations as to why most instances of detected epistasis occur at non-QTL-associated locations. The first is that there are more possible non-QTL-associated genomic locations that could serve as epistatic loci or hubs. The rat GWAS study identified 18 putative QTL. Therefore, only 360Mb of the *R. norvegicus* genome would be considered QTL-associated under our pruning and categorization strategy. The *R. norvegicus* genome assembly used in this study is approximately 2.6 Gb in size [69], meaning that there are more possible non-QTL-associated regions we could have detected in this study compared to the 18 QTL-associated regions. The mouse GWAS identified four QTL for BMI, three of which mapped to a genomic location, representing only 30Mb of the mouse genome. This same reasoning as to why there are more non-QTL-associated epistatic loci can be applied to why we observe so many more interchromosomal epistatic pairs compared to intrachromosomal pairs. Simply, there are more possible pairwise combinations that can occur across chromosomes than can occur within the same chromosome.

The second explanation is that main effects derived from significant GWAS summary statistics are not adequate predictors of epistasis. 82.4% and 82.0% of unique epistatic loci are non-QTL-associated under Cartesian and XOR models, respectively in rats. Furthermore, the dataset of SNPs in mice are not selected based upon GWAS summary statistics. Yet, there are still significant levels of statistical epistasis detected across the 19 mouse autosomes. In this regard, the mouse dataset serves as a more pertinent example of why searching for epistasis solely based upon main effects may not be optimal. Even though, to test our methodology and to compare to relevant findings in the literature, we prune the original rat dataset using GWAS *p*-values, we suggest implementing alternative pruning strategies, perhaps based on expert knowledge, allele frequency, or biological function, to test for epistasis.

Another alternative explanation is our pruning strategy itself. Our initial step is to remove redundant pairs within 10Mb upstream or downstream from each other and only the most significant pair (lowest *p*-value) is retained. In rats, there are more redundant pairs involving loci in LD with putative QTL compared to non-QTL-associated pairs because our dataset is derived from the 10,000 most significant loci in terms of GWAS *p*-values (S1 File). Perhaps with a different pruning strategy, QTL-associated loci would have possibly been highlighted more. However, our pruning strategy serves to control for high levels of LD and highlight loci with non-significant main effects as centers for epistasis. It is plausible that combinatory mutations in multiple loci, interacting in networks or pathways, may be required to explain much of the variation observed in phenotypes as canalized systems are likely resistant to alterations to one or few loci [16–19]. Thus, it is possible that we detect loci underlying BMI in *R. norvegicus* and *M. musculus* that would otherwise go undetected if only main effects are considered. Examples from our results in *rats* include the gene *Pdgfrl*, which the largest Cartesian non-QTL associated hub (chr16.54146824), is centered on and *Stk17b*, which is located just upstream of the largest XOR non-QTL-associated hub. *Pdgfrl* is a platelet-derived growth factor receptor-like gene that has been implicated in certain cancers in humans [70, 71] and *Stk17b*, serine/threonine kinase, has been associated with signal transduction and apoptosis [72, 73]. In mice, the second largest XOR non-QTL-associated hub (chr14.33090575) is located within the *Arhgap22* gene. This gene is expressed in all mammals and has been implicated as a cytoskeletal and cellular transcription regulator [74, 75] as well as being involved in the central nervous system [76]. These loci were undetected by their associated univariate analyses and this study provides evidence that their roles in BMI should be further investigated. Additional to this point, the sheer number of loci involved in significant two-way interactions compared to the number of loci with significant main effects detected in the original GWAS studies illustrate the potential importance of epistasis in understanding the genetic variance underlying complex biological traits like BMI in two closely-related species.

### Numerous epistatic events are specific to one interaction model

Although the number of epistatic loci we detect under both Cartesian and XOR models is comparable and generally overlaps in rats, there are more significant pairs reaching significance under the XOR model compared to Cartesian. These pairings are mostly distinct with only 16 pairs shared between the models. In mice, there is only one significant Cartesian pair that reached significance. This highlights that, at least in mice, using a single interaction model (in this case, Cartesian) may not uncover substantial levels of epistasis and/or lead to the assumption that no epistasis underlies the BMI phenotype in this species. Furthermore, in rats, XOR hubs are larger and more numerous than Cartesian hubs. We also observe similar results in our 3-way experiment in rats with XOR hubs being larger and triplets being mostly unique between models. Taken together, these results suggest that interaction model is an important consideration when investigating epistatic events in biological systems and that different systems and phenotypes may exhibit epistasis only under certain interaction models.

The high amount of overlap between epistatic loci is partly attributable to re-using Cartesian hub identifications for XOR epistasis and our conservative pruning and identification strategy. Despite this, the ways in which these loci interact are mostly distinct in rats. This can be explained by the many possible ways in which epistasis can theoretically occur, which Li and Reich illustrate in their enumeration of possible full penetrance models [46]. For two-way interactions, the Cartesian model of epistasis is multiplicative where the slope of one MLG is zero while another is double that of an intermediate MLG (S2 File). As we have shown, interactions following this model are indeed statistically possible and reach significance. However, genetic systems can be complex, leading to an array of possible interaction mechanisms, like in XOR (S2 File). Li and Reich only highlight full penetrance functions in their work because of the infinite possibilities of partial penetrance functions. We show here that these full penetrance functions serve as adequate models for detecting non-linear/non-additive interactions, even in continuous phenotypes (BMI) despite the binary, discrete outcomes (commonly a disease phenotype) modeled by full penetrance. Further, the rat dataset is pruned initially by main effect while the mouse dataset is not. Main effect pruning likely biases interactions towards Cartesian as strong main effects somewhat contradict the non-linearly separable MLG assumptions of the full penetrance XOR model. Despite this, we are still able to elucidate epistasis in the rat system using the XOR model with it uncovering more interactions than Cartesian in both our two-way and three-way experiments. Additionally, almost all epistasis in our mouse dataset, not pruned by main effects, are described with the XOR model. This may be evidence suggesting that XOR interactions are more abundant in loci that do not show strong main effects, however additional research is required to bolster this claim. Taken together, we provide evidence that it may not be possible for the Cartesian interaction model alone to describe the many types of epistatic relationships possible among loci, even those with strong main effects. Indeed, our results in mice suggest that the Cartesian model may not be suitable to detect any epistasis in that system for BMI. While this is also likely to be true for the XOR model in other systems and phenotypes, our results illustrate that XOR is a viable model for epistasis in two systems. This statistical evidence may justify experimental work to validate that biological interactions following an XOR-like model can evolve and be maintained in natural systems. The application of other interaction models to determine the degree of overlap between interaction models using our methodology may also warrant further investigation. It is likely that the XOR model, as with the Cartesian model, can only detect a portion of all epistatic events occurring across a genome.

### Different interaction models yield biologically-relevant enrichment in rats and mice

In rats, functional annotation of gene models 1Mb upstream and downstream of the 16 loci unique to the Cartesian model are not enriched for any GO terms and only one KEGG pathway (Ribosome). However, gene models near the 25 loci unique to the XOR model yield enrichment results primarily associated with metabolism and catalysis. Of these enrichments, a KEGG pathway of primary interest is the glucagon signaling pathway as glucagon-like-protein-1 receptor agonists (GLP-1 RAs) are emerging as important medications to lower plasma glucose and induce weight loss in Murine models and humans [77, 78]. SNPs near the genes that enriched this pathway are not implicated in the associated GWAS and could present possible targets for further study with additional experimentation.

When we investigate enrichment in the 75 epistatic loci shared between Cartesian and XOR models and the 18 putative QTL from the GWAS, immunity-related processes make up most enriched GO terms and pathways. In mice, XOR-specific GO term enrichments are mostly centered around immunity, with some notable metabolic exceptions. Since there are no Cartesian-specific epistatic loci in mice, we could not investigate Cartesian-specific enrichment. However, one of the two loci shared between models, chr12.7079769, is in close proximity to the *ApoB* gene. *ApoB* is an apolipoprotein and thus a structural component involved with the formation and synthesis of low density lipoproteins [79]. It has been shown that heterozygous mice for a knockout in *ApoB* are protected against diet-induced hypercholesterolemia after being fed a diet rich in fat and cholesterol [79]. Further, in a more recent study, disruption of *ApoB* leads to high incidences of non-alcoholic fatty liver disease [80]. Variants in this gene are likely linked to changes in cholesterol/lipid metabolism and BMI.

In rats, the XOR model uncovers an enrichment signal for metabolism that would have been missed if only the Cartesian model was applied. In contrast, in mice, strong metabolic signal is captured using the Cartesian model. However, numerous examples of epistasis and notable metabolic-related enrichments are also detected with XOR. It is important to note that because so many XOR-specific epistatic loci are discovered in mice, enrichment becomes more challenging as many gene models are considered, potentially weakening enrichment signals. It is difficult to extrapolate if a similar enrichment of immunity across shared loci, as we observe in rats, would have occurred if more epistasis was detected using the Cartesian model in mice. More experiments across breeding designs, phenotypes, and species are needed to better understand if certain interaction models are associated with specific biological processes. Taken together, however, all of these results point to the importance of using multiple interaction models when investigating epistasis.

Although links between immunity and obesity-related phenotypes have been well-documented [81–86], immunity-related enrichments and associations are commonly reported across diverse taxa and phenotypes [84, 87–90]. This is largely due to the inherent complexity of gene networks underlying immune systems across animal taxa [91], not excluding invertebrates [87, 89, 90]. Additionally, and perhaps even more importantly, genes related to immunity are likely core to general stress responses in all forms of life [82, 84, 88]. Since most phenotypes of interest to biologists and clinicians center around extreme perturbations from homeostasis (i.e., stressful conditions), it may be common to see the over-representation of immunity-related GO terms in functional annotations across biology and medicine. Our results may suggest that, in rats, shared epistatic loci between interaction models capture the dynamic and feedback-regulated nature of immunity observed in biological systems. Yet, in a closely-related species (*M. musculus*), XOR-specific epistatic loci uncover a mostly immunity-related signal, again with notable metabolic exceptions. An important consideration is that the mice and rats used in the associated experiments, despite being closely-related biological systems, were reared in different environments, were exposed to unique stressors, ate different diets, and came from different pedigree structures. Although this can serve as a limitation, one plausible explanation for the difference in enrichment profiles is the role of gut microbiota in the association between immunity and obesity. There is strong evidence in the literature of the link between the gut microbiome and immunity in affecting obesity-related phenotypes [92–95]. Furthermore, there have been notable differences identified between rats and mice concerning the role of the microbiome in immunity and obesity [96, 97]. It may be possible that our distinct enrichment profiles between systems may be highlighting species-level differences in how obesity is related to immunity. Alternatively, we could also be capturing signals associated with differences between methodologies and/or environments. Additional experiments are required to elucidate the differences in how epistatic interactions underlie obesity in these species.

In rats, the XOR model identifies epistatic loci enriched for more biologically relevant functions and processes (metabolism and BMI). This may be surprising as full penetrance XOR logic may not be biologically plausible due to genetic constraints. However, we have shown that a full penetrance XOR model is adequate to detect statistical epistasis in two systems. A possible scenario in which XOR-like epistasis may occur is during gene regulation. An illustrative example could be when the presence of one activator (A), encoded by gene one, while in the presence of another activator (B), encoded by gene two, results in the transcription of gene three. However, in the presence of both activators, gene three is not expressed. This could be due to activators A and B binding to one another when they co-occur, inhibiting their DNA binding motifs, or because both activators collectively block other transcription enzymes from binding [48]. Mechanistic examples of XOR interactions in transcriptional regulation such as this may be plausible. However, examples of XOR logic in other biological processes may seem less likely. The XOR model assumes a phenotypic score in one extreme when only one locus is in a heterozygous state but the other extreme if both loci are heterozygous (S2 File). In terms of BMI, where higher phenotypic scores are deleterious in a static environment where food is abundant, this would translate to heterozygote disadvantages in one locus when the other locus is in a homozygous state. However, if both loci are in a heterozygous state, then the MLG is associated with lower BMI, leading to a heterozygote advantage. Heterotic relationships are commonly described in natural systems, primarily in crop plants [98] but also in humans as observed in the genetic mechanisms underlying sickle-cell anemia and malaria resistance [99]. Yet, the variable phenotypic diversity of sickle cell morphology and/or malaria resistance violates Mendelian expectations [11]. It is possible that one explanation for these abnormalities are epistatic interactions between loci, including relationships described by interaction models other than Cartesian. Exploring these phenotypes with multiple interaction models may assist in explaining deviations from Mendelian expectations in malaria and other phenotypes.

## Conclusions

Regardless of the underlying mechanisms, our results from rats illustrate that many epistatic loci are located near genes that are enriched for metabolic functions and interact in a manner only detectable by the XOR model. In our mouse samples, we illustrate that most epistatic events underlying BMI only reach significance using the XOR model. However, it is important to note that considerable epistasis is uncovered in rats using the Cartesian model that has an epistatic landscape distinct from that of XOR’s and important, albeit limited, biologically-relevant loci are detected using the Cartesian model in mice. Thus, our results suggest that epistatic loci are detected and interact in different ways depending on the interaction model used. The latter suggests that distinct interaction mechanisms may exist for different biological networks involving shared loci.

Our study has been illustrative in showing that epistatic interactions in biological systems are likely far more complex and ubiquitous than previously thought. This necessitates the consideration of different models of interaction for investigating epistasis. The algorithms we have given provide tools for collecting statistical evidence for epistasis using different interaction models. The matrix-based permutation testing algorithm we have presented can give further statistical evidence and can also be simplified and applied in GWAS or eQTL studies.

In the future, we suggest applying our methodology to diverse taxa and phenotypes to investigate the complexity of epistasis and warrant the development of validation studies to describe biological interactions. We hope our methodology helps to further elucidate complex traits, including many human diseases, by uncovering genetic relationships that have thus far been elusive to standard analyses. Extending the algorithms given for logistic regression for case/control studies and generalized linear models would be very useful and important especially for handling population structure directly. Li and Reich presented many different models for epistasis and corresponding penetrance functions [46]. A natural extension to this work would be to use additional penetrance functions for modeling interactions. This can already be done with the algorithms given with the only change being how the interaction terms are encoded according to the models. The work for this software extension is already under development.

### Supplementary Information

**Additional file 1.** S1 File. Computation time results, simulated epistasis results, phantom epistasis results, and raw and final data for Pairwise and 3-way Epistasis in rats. The first data tabs have results for the computation time, simulated epistasis, and phantom epistasis experiments. Next, this file also contains all significant pairs and triplets found for XOR and Cartesian models in rats and corresponding counts for loci before and after pruning and mapping strategy was applied. It also contains the raw pair results for the XOR model using permutation test. Full data for the GO and KEGG pathway analysis in rats is also included.

**Additional file 2.** S2 File. Penetrance Functions and Additional Experiments. This file contains examples of theoretical penetrance functions and the sample penetrance functions from loci in the mouse and rat data sets. In addition, this file contains results from correlation tests in rats between GWAS summary statistics and detected epistatic events in rats and GO-term enrichment dotplots for GWAS QTLs in rats and mice.

**Additional file 3.** S3 File. Raw and final data for Pairwise and 3-way Epistasis in mice. This file contains all significant pairs and triplets found for XOR and Cartesian models in mice and corresponding counts for loci before and after pruning and mapping strategy was applied. It also includes the GO and KEGG pathway analysis for the loci in epistasis after mapping and pruning in mice.

## Data Availability

Rat phenotype data and GWAS summary statistics are available at https://library.ucsd.edu/dc/object/bb83725195. Rat genotype data are available at https://library.ucsd.edu/dc/object/bb15123938. Mouse genotype and phenotype data are available via the “BGLR” package in R. The implementations of these algorithms given in Python and code for various experiments in Python and R are offered via GitHub at https://github.com/EpistasisLab/epistasis_detection.

## Abbreviations

BMI: Body mass index
bp: Base pair(s)
Mb: Megabase(s) (1*e*6 base pairs)
Cart: Cartesian
eQTL: Expression quantitative trait loci(us)
FDR: False discovery rate
GO: Gene ontology
GRM: Genetic relatedness matrix
GWAS: Genome-wide association study
KEGG: Kyoto encyclopedia of genes and genomes
LD: Linkage disequilibrium
MAPIT: MArginal ePIstasis Test
MDR: Multi-factor dimensionality reduction
MLG: Multi-locus genotype
MSE: Mean square error
QTL: Quantitative trait loci(us)
SNP: Single nucleotide polymorphism
XOR: eXclusive OR
